# Combination of genetic analysis and ancient literature survey reveals the divergence of traditional *Brassica rapa* varieties from Kyoto, Japan

**DOI:** 10.1038/s41438-021-00569-0

**Published:** 2021-06-01

**Authors:** Yaichi Kawakatsu, Tomoaki Sakamoto, Hokuto Nakayama, Kaori Kaminoyama, Kaori Igarashi, Masaki Yasugi, Hiroshi Kudoh, Atsushi J. Nagano, Kentaro Yano, Nakao Kubo, Michitaka Notaguchi, Seisuke Kimura

**Affiliations:** 1 Faculty of Life Sciences, Kyoto Sangyo University, Kamigamo-motoyama, Kita-ku, Kyoto, 603-8555 Japan; 2 Bioscience and Biotechnology Center, Nagoya University, Furo-cho, Chikusa-ku, Nagoya, 464-8601 Japan; 3 Center for Plant Sciences, Kyoto Sangyo University, Kamigamo-motoyama, Kita-ku, Kyoto, 603-8555 Japan; 4 Graduate School of Science, The University of Tokyo, 7-3-1 Hongo, Bunkyo-ku, Tokyo, 113-0033 Japan; 5 School of Agriculture, Meiji University, 1-1-1, Higashi-Mita, Tama-ku, Kawasaki, Kanagawa 214-8571 Japan; 6 Faculty of Engineering, Utsunomiya University, 7-1-2 Yoto, Utsunomiya, Tochigi, 321-8585 Japan; 7 Center for Ecological Research, Kyoto University, Hirano 2-509-3, Otsu, Shiga, 520-2113 Japan; 8 Faculty of Agriculture, Ryukoku University, 1-5 Yokotani, Seta Oe-cho, Otsu, Shiga 520-2194 Japan; 9 Graduate School of Life and Environmental Sciences, Kyoto Prefectural University, 1-5 Hangi-cho, Shimogamo, Sakyo-ku, Kyoto, 606-8522 Japan; 10 Biotechnology Research Department, Kyoto Prefectural Agriculture, Forestry and Fisheries Technology Center, 74 Oji, Kitainayazuma, Seika-cho, Soraku-gun, Kyoto, 619-0244 Japan; 11 Graduate School of Bioagricultural Sciences, Nagoya University, Furo-cho, Chikusa-ku, Nagoya, 464-8601 Japan; 12 Institute of Transformative Bio-Molecules, Nagoya University, Furo-cho, Chikusa-ku, Nagoya, 464-8601 Japan

**Keywords:** Plant breeding, Plant hybridization

## Abstract

Since ancient times, humans have bred several plants that we rely on today. However, little is known about the divergence of most of these plants. In the present study, we investigated the divergence of Mibuna (*Brassica rapa* L. subsp. *nipposinica* L. H. Bailey), a traditional leafy vegetable in Kyoto (Japan), by combining genetic analysis and a survey of ancient literature. Mibuna is considered to have been bred 200 years ago from Mizuna, another traditional leafy vegetable in Kyoto. Mibuna has simple spatulate leaves, whereas Mizuna has characteristic serrated leaves. The quantitative trait loci (QTL) and gene expression analyses suggested that the downregulation of *BrTCP15* expression contributed to the change in the leaf shape from serrated to simple spatulate. Interestingly, the SNP analysis indicated that the genomic region containing the *BrTCP15* locus was transferred to Mibuna by introgression. Furthermore, we conducted a survey of ancient literature to reveal the divergence of Mibuna and found that hybridization between Mizuna and a simple-leaved turnip might have occurred in the past. Indeed, the genomic analysis of multiple turnip cultivars showed that one of the cultivars, Murasakihime, has almost the same sequence in the *BrTCP15* region as Mibuna. These results suggest that the hybridization between Mizuna and turnip has resulted in the establishment of Mibuna.

## Introduction

Generally, traditional indigenous vegetables are native varieties that have been grown or cultivated in a given region for a long time, and many of them have characteristic traits. During recent years, traditional vegetables have garnered attention owing to their regional revitalization and importance in maintaining crop biodiversity. To maintain and effectively use such vegetables, an in-depth understanding of their origin and breeding history is needed. However, the divergence of most of these traditional vegetables has not been well described to date.

Kyoto was the capital of Japan from 794 to 1868, when many varieties of vegetables were gathered and cultivated as offerings to the emperor^1,2^. Consequently, there are several traditional vegetables known as “*Kyo-yasai*” (“*Kyo*” and “*yasai*” mean Kyoto and vegetables, respectively) in the Kyoto region. Several types of *Kyo*-*yasai* are still being actively cultivated, including varieties of turnip (*Brassica rapa* L.), radish (*Raphanus sativus* L.), eggplant (*Solanum melongena* L.), pumpkin (*Cucurbita moschata* Duch.), and various other leafy vegetables^2^.

**Fig. 1:**
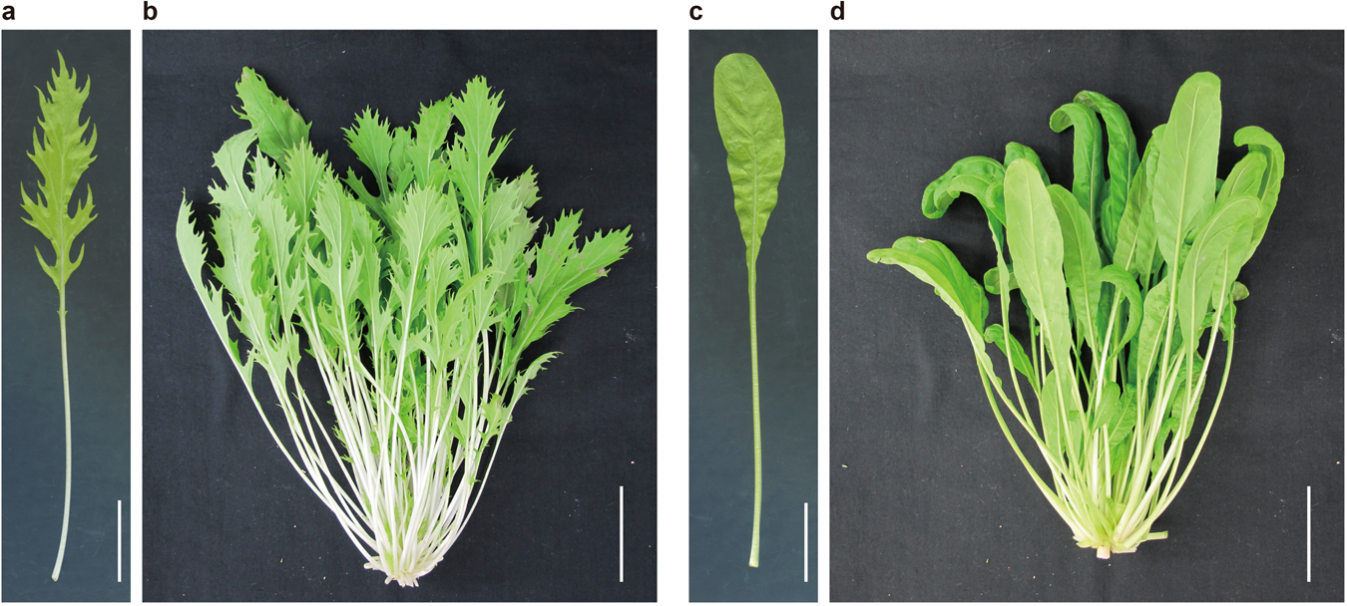
Leaf morphology of Mizuna and Mibuna. **a** Mizuna leaf grown for 4 weeks and **b** plant habit grown for 3 months. **c** Mibuna leaf grown for 4 weeks and **d** plant habit grown for 3 months. Scale bars: (**a**, **c**) 3 cm, (**b**, **d**) 5 cm

Among the types of *Kyo*-*yasai*, Mizuna and Mibuna (*B. rapa* L. subsp*. nipposinica* L. H. Bailey) are particularly popular leafy vegetables (Fig. 1). Mizuna was used to be eaten boiled, whereas nowadays, it is often used in salads. Mibuna has a harder texture and stronger spiciness than Mizuna and is often used in a pickled form. The most conspicuous morphological difference between these varieties is their leaf shape. Mizuna has characteristic serrated leaves (Fig. 1a, b), whereas Mibuna has simple spatulate leaves (Fig. 1c, d). Phylogenetic analyses of *B. rapa* subspecies have suggested that Mizuna and Mibuna form distinct subgroups and are unique to Japan^3–5^. Considering the economic importance of *B. rapa* crops, their relationships and order of domestication have to be understood. In fact, several *B. rapa* crops have been investigated by phylogenetic and population genetics analyses^6,7^. Mizuna and Mibuna are intriguing targets to gain insights into the emergence of local-subspecies.

**Fig. 2:**
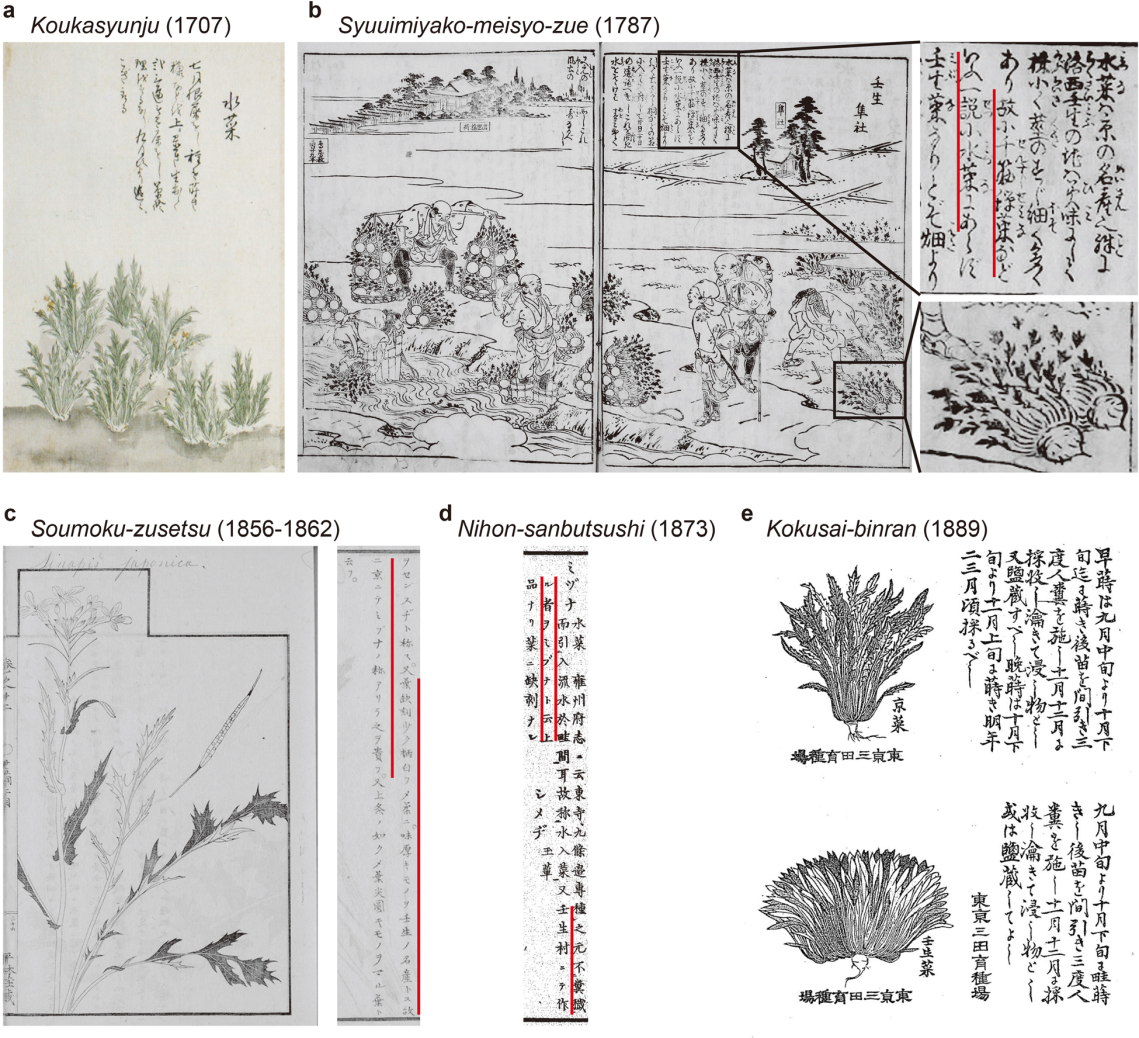
Literature sources in which Mizuna and/or Mibuna are depicted and their publication years. **a** Picture of Mizuna depicted in *Koukasyunju*, published in 1707. **b** *Syuuimiyako-meisyo-zue*, published in 1787. Text at the top of the picture (upper right) and the vegetables drawn in the back (bottom right) are enlarged. The text in red can be translated as “In one theory, it is Mibuna, not Mizuna”. **c** The picture of Mizuna (left) and description of Mibuna in *Soumoku-zusetsu* published in 1856–1862. The text in red can be translated as “Mibuna is a product of Mibu region with a few leaf serrations, white petioles, and a strong taste”. **d** Description of Mizuna in *Nihon-Sanbutsushi*, published in 1873. The text in red lines can be translated as “The special product made in Mibu region is called Mibuna, and there is no serration on the leaves”. **e** Pictures of Mizuna (top) and Mibuna (bottom) from *Kokusai-binran*, published in 1889. The red lines were added by us

Mizuna and Mibuna are among the oldest vegetables cultivated in Kyoto and are often described in old Japanese literature. Previously, we surveyed ancient literature mentioning Mizuna and Mibuna to investigate when and how these vegetables were developed, focusing on the leaf shape^8^. The earliest reference that contains a picture of a Mizuna is *Koukasyunju*, an agricultural book published in 1707^8^. Mizuna depicted in this book had almost the same form as that of the present-day Mizuna (Fig. 2a). The earliest picture of Mibuna is presented in *Syuuimiyako-meisyo-zue*, a travel guide published in 1787^8^, and the plant in the picture has serrated leaves similar to those in the present-day Mizuna (Fig. 2b). *Soumoku-zusetsu*, a botanical picture book published in 1856–1862^8^, describes that “Mibuna has less-serrated leaves than Mizuna” (Fig. 2c). This description suggests that at that time, Mibuna showed an intermediate phenotype between current Mizuna and Mibuna; current Mibuna has non-serrated leaves rather than slightly serrated leaves. *Nihon-sanbutsushi*, a handbook for local products published in 1873,^8^ states “Mibuna leaves have no serrations,” indicating that establishment of spatulate leaves in Mibuna occurred during this time (Fig. 2d). Later, *Kokusai-binran*, a handbook for crops and vegetables published in 1889^8^, included separate drawings of Mizuna with serrations and Mibuna without serrations (Fig. 2e). Based on this literature survey, we concluded that Mibuna diverged from Mizuna in the late 1800s. However, the cause or genetic basis for the above-described change in leaf shape is completely unknown.

Serration is one of the major leaf traits, and it is controlled by several genes. TEOSINTE BRANCHED/CYCLOIDEA/PCF (TCP) is a transcription factor that contains a bHLH motif, and it promotes plant development and controls cell proliferation^9–11^. In *Arabidopsis thaliana*, double mutants of *TCP14* and *TCP15* are characterized by broader leaves than those of WT^10^, and *TCP15* overexpression causes the development of serrated leaves^12^. The *TCP* family genes also control the leaf morphology of other plant species, such as tomatoes (*Solanum lycopersicum* L.), lettuce (*Lactuca sativa* L.), and snapdragon (*Antirrhinum majus* L.)^13–16^. PINFORMED1 (*PIN1*) is an auxin transporter gene that contributes to the formation of marginal serrations in leaves^17^. ASYMMETRIC LEAVES2/LATERAL ORGAN BOUNDARIES (*AS2/LOB*), which encodes the AS2/LOB domain, is involved in the development of symmetric flat leaf laminas^18,19^. AUXIN RESPONSE FACTOR 3/ETTIN (*ARF3/ETT*) and *ARF4* are transcription factors essential for the establishment of abaxial identity initiated by *KANADI*^20–22^. *ATHB13* is a member of the homeodomain leucine zipper (HD-Zip) transcription factor family, which is ectopically expressed in altered leaf development, specifically on sugar-containing media^23^.

As *B. rapa* includes different kinds of cultivated plants, genetic analyses, such as QTL analysis, gene mapping, and genome-wide analysis, have been carried out to study this species. The genetic basis of *B. rapa* traits such as head formation^24–27^, flowering time^28–30^, trichome formation^24,31,32^, and seed coat color^32^ has been investigated. An analysis of leaf morphology of *B. rapa* showed that the *TCP* genes are involved in leaf bulge and head formation^25^. In addition, a leaf lobe-related gene has been identified on LG A10^30,33^, and a homolog of LATE MERISTEM IDENTITY 1 (*BrLMI1*) is considered a potential candidate associated with leaf lobe development^34^. Furthermore, next-generation sequencing (NGS) technology enables rapid and high-throughput QTL detection. Restriction site-associated DNA sequencing (RAD-seq) is a method for identifying QTLs by combining NGS with RAD markers, which are short fragments of DNA adjacent to a particular restriction enzyme recognition site^35^. QTL-seq is a method used for whole-genome resequencing of DNA from two populations with different characteristics^36^.

In the present study, we investigated the divergence of Mibuna, focusing on leaf shape change, using QTL, RAD-seq, and QTL-seq analyses. Among the candidate genes identified in these analyses, the genes responsible for the variation in leaf shape between Mizuna and Mibuna were detected by comparing their expression levels using RNA-seq. In addition, we conducted a survey of ancient literature to further estimate how these varieties emerged in Kyoto.

## Results

### Emergence of Mibuna with spatulate leaves occurred in the late 1800s, and the main responsible gene is located on LG A07

**Fig. 3:**
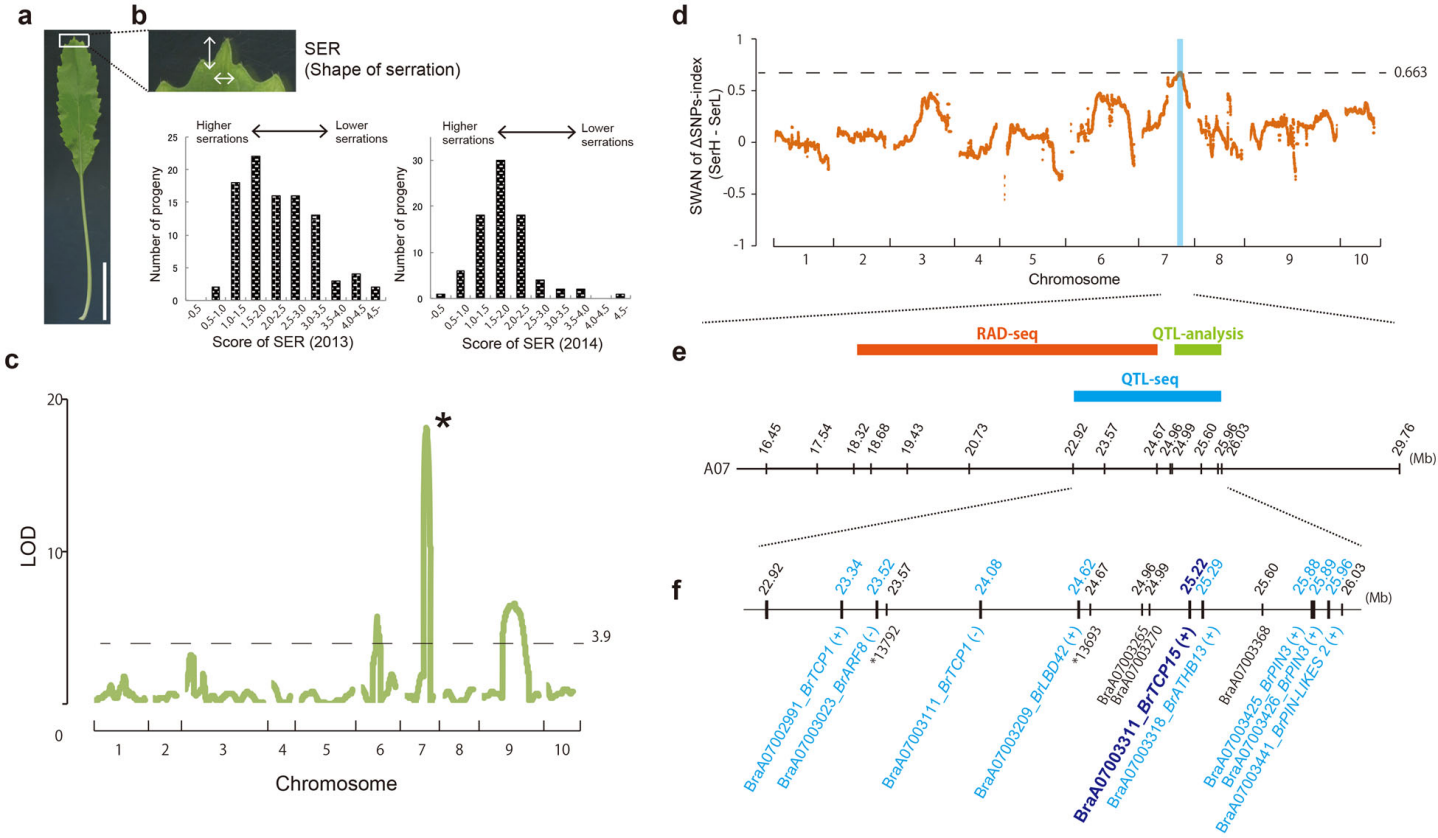
Leaf shape of the F_1_ generation of Mibuna and Mizuna, and the results of the QTL and QTL-seq analyses of these F_2_ generations. **a** Leaf shape of the F_1_ plants of Mibuna and Mizuna. Scale bar; 3 cm. **b** Enlarged view of F_1_ leaf tip and frequency distribution of SER score in the F_2_ generation of Mizuna and Mibuna in 2013 (left) and 2014 (right). White arrows indicate the height and width of the serration at the tip. **c** Results of the QTL analysis of leaf serration. The horizontal and vertical axes show the position of the chromosome and the LOD score, respectively. The asterisk indicates the A07 QTL with the highest contribution. The dotted line (3.9) shows the threshold value. **d** Results of the QTL-seq analysis. The horizontal axis represents the chromosome position. The dotted line is the upper limit of the outlier (0.663) and the blue area indicates the area over this line. **e** Enlarged view of the QTLs. The QTLs are shown above the map, and the numbers on the map are the locations of the gene and marker (Mb). (+) or (−) indicates the direction of the gene. **f** Detailed map of the QTL-seq locus. The blue and black letters indicate the gene and marker names, respectively. The marker positions (Mb) are listed above the map

Based on the description in ancient literature (Fig. 2b), we estimated that the leaf form of Mibuna was established ~200 years ago. To reveal the genetic basis of the change in leaf shape from Mizuna to Mibuna, genetic analysis was performed. The leaf morphology of the F_1_ generation produced by crossing Mibuna and Mizuna showed an intermediate phenotype with small serrations (Fig. 3a). For the genetic analyses, the score of the height of serration (SER) was quantified from the serration at the leaf tip by dividing its width by its height (Fig. 3b). The average SER scores of Mizuna and the F_1_ plants were 0.51 and 2.1, respectively, whereas the score of Mibuna could not be obtained because of the lack of serrations. The SER score of the F_2_ plants did not show any typical discrete segregation, indicating that multiple loci contribute to the SER trait (Fig. 3b).

We then performed QTL and RAD-seq analyses of the F_2_ generations sampled in 2013 and 2014, respectively. The QTL analysis showed that the QTLs that contributed to the SER were located on LG A06, A07, and A09 with contribution rates of 8.7%, 37.9%, and 13.2%, respectively (Fig. 3c and Supplementary Table S1). The RAD-seq analysis showed that the QTLs for the same trait were located on LG A06 and A07, with contribution rates of 24.7% and 40.3%, respectively (Supplementary Table S1 and Supplementary Fig. S1). These results were consistent with the expected number of QTLs from the phenotypic distribution of the F_2_ population. These results suggest that the QTL with the highest contribution is located on LG A07.

To identify the genetic locus that contributes to serrations in detail, QTL-seq analysis was performed. Whole-genome sequencing of the F_2_ population with higher (SerH) and lower SER scores (SerL) was performed, and the SNP-index, that is, the ratio between the number of reads of SNP in each population and the total number of reads corresponding to the SNP^36,37^, was detected. To compare the two SNP indices, the ΔSNP-index (SerH-SerL) was calculated by subtracting each SNP-index value of the SerL from that of the SerH population^36^. The ΔSNP-index is 1 if all the short reads represent the genome from the low serration parent, that is, Mibuna. To facilitate graph visualization, sliding window analysis (SWAN) was performed by taking the average ΔSNP-index in a given genomic interval^36^. From this analysis, we detected a locus of ~3 Mbp located between 22,915 and 26,030 kbp of LG A07, where the SWAN score was higher than the outlier of the ΔSNP-index (Fig. 3d). This locus was close to the QTLs detected by QTL and RAD-seq analyses (Fig. 3e). The responsible gene was thought to be located in this locus.

The 3-Mbp locus of LG A07, detected by QTL-seq analysis, contained 530 genes (Supplementary Table S2). Among them, nine are homologous genes; they are known to be involved in leaf morphogenesis in *A. thaliana*^9,10,17–19,21–23^. These genes were considered as candidate genes (Fig. 3f and Supplementary Table S2). No SNPs were detected in the coding regions of these nine genes, which altered the amino acid sequence between the SerL and SerH populations, suggesting that the genes responsible for leaf shape variation show different expression patterns between Mizuna and Mibuna.

### Downregulation of *TCP15* homolog expression might be involved in the leaf shape change

**Fig. 4:**
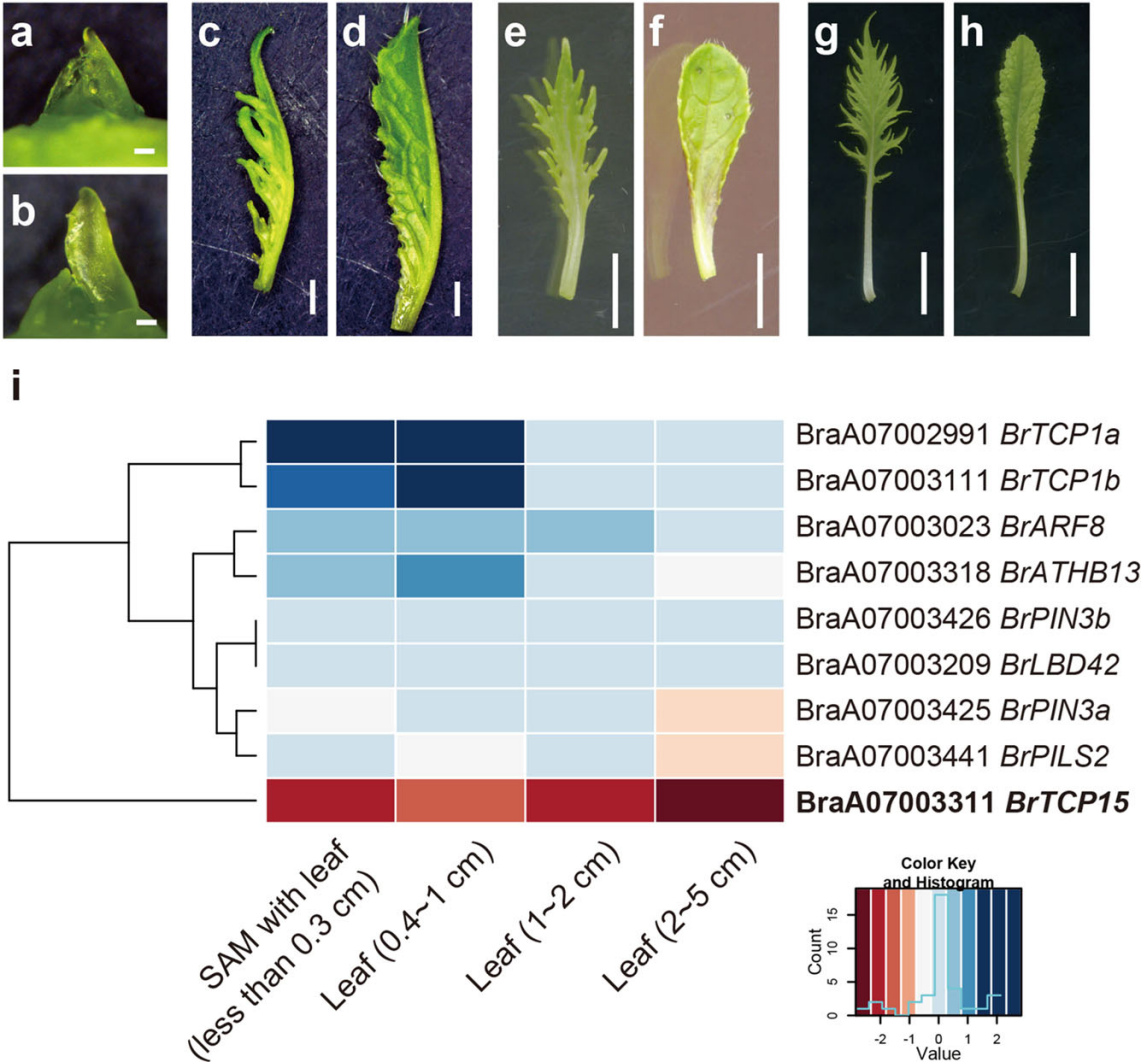
RNA-seq analysis of A07 candidate genes. **a–h** Leaf morphology of Mizuna (**a**, **c**, **e**, and **g**) and Mibuna (**b**, **d**, **f**, and **h**) grown for 3 weeks after seeding. The organs are SAM with leaf of lengths under 0.3 cm (**a**, **b**: scale bar 100 µm), 0.3–1 cm (**c**, **d**: scale bar 1 mm), 1–2 cm (**e**, **f**: scale bar 5 mm), and 2–5 cm (**g**, **h**: scale bar 1 cm). **i** Heat map of the log FC (Mibuna/Mizuna) using RNA-seq. Blue boxes indicate higher expression in Mibuna and red boxes indicate higher expression in Mizuna

To identify genes responsible for the diversity in leaf shape morphology between Mizuna and Mibuna, an RNA-seq analysis of four kinds of tissues was performed (Fig. 4a–h). Among the nine candidate genes, BraA07003311 (*BrTCP15*), BraA07002991 (*BrTCP1a*), and BraA07003111 (*BrTCP1b*) showed absolute values of log FC (Mibuna/Mizuna) of >2 at some stage of development (Fig. 4i and Supplementary Fig. S2a). Among these *BrTCPs*, only *BrTCP15* showed higher expression in Mizuna than in Mibuna, and the absolute values of log FC (Mibuna/Mizuna) was >1.5 at all developmental stages (Fig. 4i). In *A. thaliana*, plants expressing *TCP15* had serrated leaves^10^, and this is consistent with our observations in Mizuna.

**Fig. 5:**
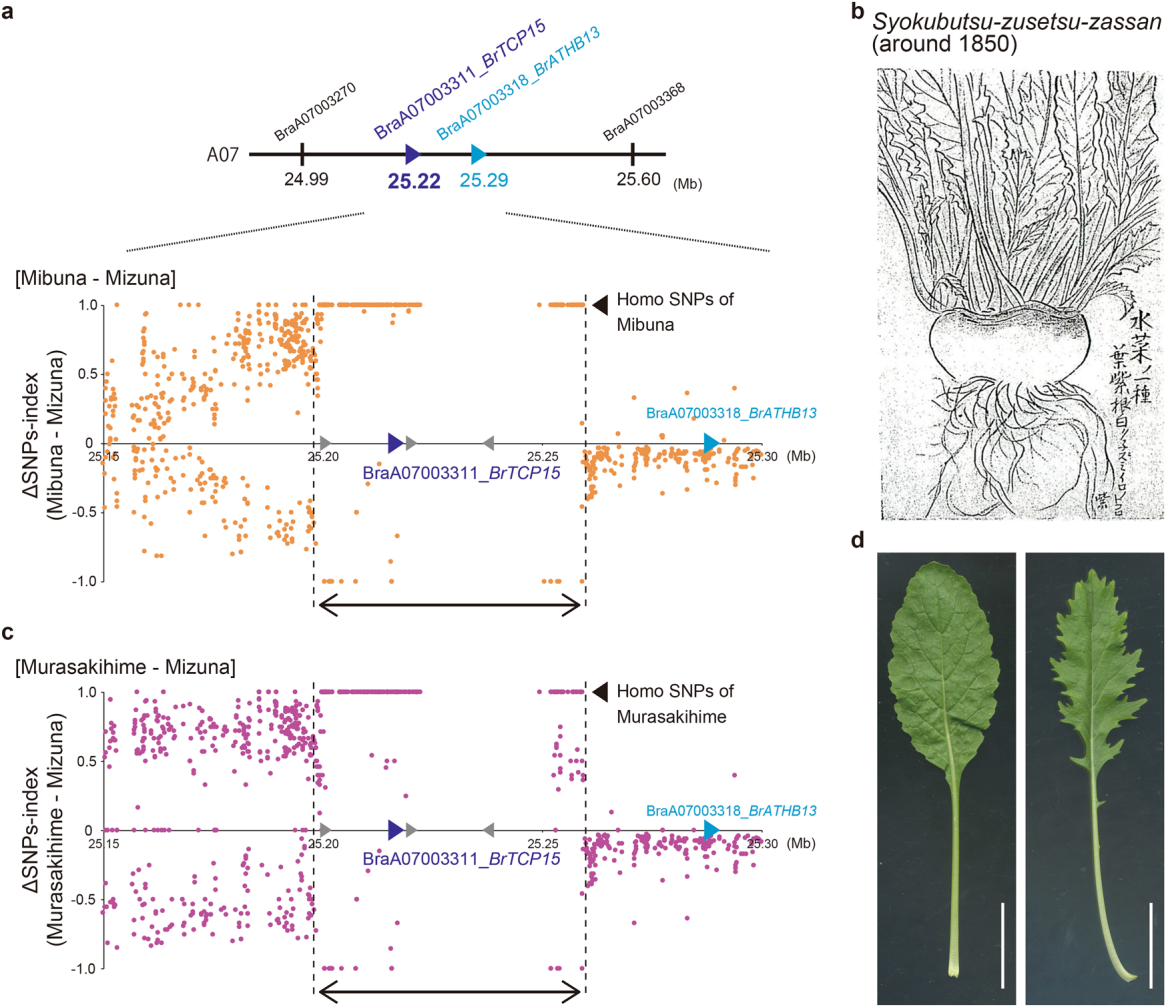
Comparison of sequences around *BrTCP15* between *Brassica rapa* varieties, along with the leaf shape of Murasakihime and a hybrid between Mizuna and Murasakihime. **a** Gene and marker positions around *BrTCP15* and graph of the ΔSNPs-index (Mibuna–Mizuna) on LG A07. The horizontal axis represents the position around *BrTCP15* on chromosome A07 (Mb). Triangles on the horizontal line show *BrTCP15* (blue), *BrATHB13* (cyan), and other genes (gray) within the locus of ~61 kbp. The dotted lines indicated by the double-headed arrow show an area of ~61 kbp between 25,198 and 25,259 kbp, in which Mibuna-allele homo SNPs are concentrated. **b** Picture of “A type of Mizuna” depicted in *Syokubutsu-zusetsu-zassan*, published around 1850. The text can be translated as “A type of Mizuna. The leaves are purple, and the root is white. The dark colored parts are purple.” **c** A graph of the ΔSNPs-index (Murasakihime-Mizuna). Triangles and dotted lines indicate the same as described in **a**. **d** The leaf shapes of turnip variety Murasakihime (left) and a hybrid of Mizuna and Murasakihime (right) grown for 4 weeks. Scale bars; 3 cm

A comparison of the genome sequence close to *BrTCP15* between Mizuna and Mibuna revealed that the Mibuna-allele homologous SNPs are concentrated in the locus of 25,198–25,259 kbp, which is ~61-kbp long (Fig. 5a). At this locus, there are 292 SNPs between Mizuna and Mibuna, and among them, 238 SNPs had ΔSNPs-index (Mibuna-Mizuna) of 1. This suggested that these 238 SNPs are homologous SNPs of Mibuna. *BrATHB13*, the candidate gene located closest to *BrTCP15*, is outside this locus (Fig. 5a). In addition, no such loci were found near *BrTCP1a* and *BrTCP1b* (Supplementary Fig. S2b). This 61-kbp chromosomal locus probably introgressed from other *B. rapa* species. From these results, we conclude that *BrTCP15* on LG A07 is the gene most responsible for leaf shape variation between Mizuna and Mibuna, and the different expression levels of *BrTCP15* are caused by gene introgression.

### Out-crossing with turnip caused the change in leaf shape in Mibuna

Based on the literature survey, we estimated the cause of the divergence between Mibuna and Mizuna. The picture of Mibuna found in *Syuuimiyako-meisyo-zue* showed that it had serrated leaves and thicker roots, similar to those in turnips (Fig. 2b)^8^. In addition, in *Syokubutsu-zusetsu-zassan*, a research note written ~1850 by Keisuke Ito, an old Japanese botanist, there is a description of a Mizuna variety (Fig. 5b). Interestingly, unlike those in present-day Mizuna, this variety has slightly serrated leaves and turnip-like roots^8^. Turnips are a subspecies of *B. rapa*. They can be crossed with Mizuna, and many old Japanese turnips varieties have spatulate leaves. Therefore, it is possible that the 61-kbp chromosomal locus may have been introgressed into Mizuna through crossing with turnips. Additionally, in *Syokubutsu-zusetsu-zassan*, the description of the vegetable states “The parts with dark ink are purple” (Fig. 5b), suggesting that the upper part of this vegetable root was purple. Thus, purple turnips were considered as an out-crossed variety.

To test the above-mentioned hypothesis, the genome sequences of five varieties of purple turnips (Murasakihime, Asuka akane, Hinona, Tsuda, and Ayameyuki), which were mostly bred around the Kyoto region, were analyzed. Among these turnip varieties, the Murasakihime variety had a locus of 61-kbp homo-SNPs similar to that in Mibuna (Fig. 5c). In this locus, Murasakihime has 198 of the same homo SNPs out of the 238 homo SNPs found in Mibuna. On the contrary, similar genome loci were not found in the other four turnip varieties (Supplementary Fig. S3). These analyses suggested that the 61-kbp chromosome locus originated from an ancestor of Murasakihime. Indeed, the F_1_ plants obtained from a cross between Mizuna and Murasakihime showed an intermediate leaf shape (Fig. 5d), which resembled that shown in the picture of a Mizuna variety from *Syokubutsu-zusetsu-zassan* (Fig. 5b).

In summary, our results suggested that the change in the leaf shape from serrated Mizuna leaf to spatulate Mibuna leaf may be a consequence of the introgression of the *BrTCP15* genomic locus from turnip into Mizuna by out-crossing, resulting in a decrease in the expression of *BrTCP15*.

## Discussion

### Mibuna, a traditional *B. rapa* leafy vegetable from Kyoto, might have arisen by natural out-crossing between Mizuna and turnip

**Fig. 6:**
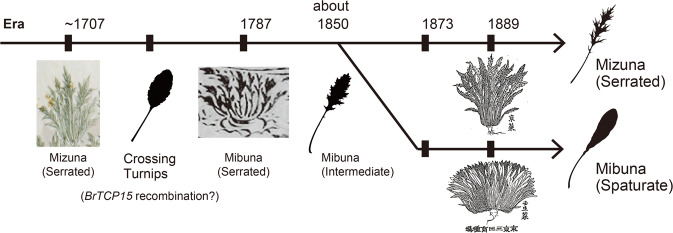
Breeding process of Mibuna inferred from literature. Diagram of the divergence of Mibuna from Mizuna, as inferred from the literature survey. The ancestors of Mizuna and Mibuna had serrated leaves similar to those of current Mizuna. Hybridization with turnip before 1787 resulted in the emergence of Mibuna with intermediate leaves. Later, in 1873, Mibuna with round leaves appeared

Plant breeding has a long history, and many useful varieties of cultivated plants have been produced by breeding. The changes in plant morphology during the breeding process can be remarkable. A previous study of *B. rapa* crops by combining phylogenetic, genetic structure, and demographic analyses comprehensively described the process of domestication in Asia and Europe^6^. These inferred times of demographic events in the history of *B. rapa* are consistent with the written records from antiquity that document these crops^6^. The present study revealed that the Japanese subspecies of *B. rapa* Mizuna with serrated leaves was crossed with turnip in the late 1800s, resulting in the development of Mibuna with spatulate leaves, and the main reason for this leaf shape change was the downregulation of *BrTCP15* on LG A07 (Fig. 4i). From the literature survey, we conclude that the divergence of Mibuna from Mizuna occurred gradually in the Mibu region of Kyoto from 1787 to 1873, with the introgression of *TCP15* from turnip (Fig. 6). Genome sequencing revealed that Murasakihime, a variety of turnip, has the same Mibuna-allele homo-SNPs around *BrTCP15* (Fig. 5c). Therefore, *BrTCP15* is considered to be recombined from a turnip cultivated in Kyoto ~1800. Because Murasakihime was bred in recent years, it is unlikely that this turnip was directly involved in the development of Mibuna. It is possible that an ancestor of Murasakihime was cultivated near the Mibu region, and Mibuna might be a result of crossing between this turnip and Mizuna. Mibuna was selected and fixed from a Mizuna population because of the attractive features, such as the spatulate leaf shape, as well as taste and texture (Fig. 2c)^8^. In this study, we identified causal underlying genetic background of the leaf shape of Mibuna, and showed that a previous cross with turnip resulted in this vegetable. In future studies, other attractive traits found in Mibuna can be addressed by analyzing the introgressed loci of turnip.

### *TCP* is the key regulatory factor for the change in leaf shape between Mizuna and Mibuna

The *TCP* family of plant-specific transcription factors regulates plant growth and leaf development by affecting cell proliferation and differentiation^11,16,38^. Previous studies have revealed that *TCP15* expressed with the 35S CaMV promoter shows serrated leaves^12^, and *TCP15* fused to the EAR (SRDX) repressor domain shows upward curling leaves^39^. These studies indicated that the expression level of *TCP15* affects leaf morphogenesis, and the upregulation of *TCP15* results in the development of serrated leaves. *TCP* was also found to contribute to leaf morphogenesis in other plants^13–16^, and in lettuce, the difference in *TCP* expression is responsible for the leaf shape variation between cultivars^14^.

In *B. rapa*, the formation of leaf lobes is influenced by QTL on LG A10^30,33^. *BrLMI1* is located on LG A10 in *B. rapa*, and a homolog of this gene *LOBED-LEAF 1* (*BnLL1*) is considered responsible for leaf morphology in *Brassica napus*^34^. On the contrary, no QTL related to serration was found on LG A10 in Mizuna and Mibuna (Fig. 3c and Supplementary Table S1), and *BrLMI1* was not found in the QTL on LG A07. Therefore, we concluded that *BrLMI1* does not contribute to the formation of serrations in Mizuna. A close observation of Mizuna revealed leaf lobes formed by large incisions in the lower half of the leaf blade, in addition to the serrations formed by fine cuts (Fig. 1a). Further investigation of this trait might reveal other causative genes for leaf morphology.

### Combination of genetic analysis and ancient literature survey is a powerful strategy for studying breeding histories of indigenous plant varieties

Traditional vegetables have several benefits for our diet and are often used as symbols of regional revitalization. For example, Kaga and Noto vegetables from Ishikawa Prefecture in Japan are registered in the Globally Important Agricultural Heritage Systems (GIAHS)^40^. Kyoto is an ancient capital of Japan, and because of the principle of market competition, several high-quality vegetables have been produced as brand vegetables in this region. During this period, because the civilization of Kyoto was highly advanced and agricultural records were maintained, the breeding process of traditional vegetables can be clarified. Our study showed that combining two different methods, genetic analysis and literature survey, can be effective for understanding the breeding history of various plants, including traditional vegetables.

## Materials and methods

### Plant materials and phenotype analysis

Seeds of Mizuna (Kyo-mizore), Mibuna (Kyo-nishiki), and turnip (Hinona, Tsuda, Ayameyuki, Murasakihime, and Asuka akane) were purchased from a nursery company (Mizuna, Mibuna, Hinona, Tsuda, and Ayameyuki seeds were obtained from Takii & Co., Ltd., Kyoto, Japan; Murasakihime seeds were obtained from Futaba Seed, Okinawa, Japan; and Asuka akane seeds were obtained from Nanto Seeds Co., Ltd., Nara, Japan). To obtain F_1_ and F_2_ generations, these seeds were sown in Jiffy pots (10 cm diameter × 8 cm height; Jiffy, Kristiansand, Norway) containing kumiai nippi soil (Nihon Hiryo Co., Ltd., Tokyo, Japan). They were cultivated for 3 weeks in a greenhouse in Kyoto Sangyo University at 23 °C. The plants were transferred into larger pots (SLITPOT CSM-150; KANEYA Co., Ltd, Aichi, Japan) containing kumiai nippi soil, vernalized for 40 days at 4 °C, and moved back to the greenhouse. After flowering, Mibuna was crossed with Mibuna, to obtain the F_1_ plants. The crossing of turnip with Mizuna was carried out in the same manner. The F_1_ plants were cultivated in the same manner as their parent plants, and the F_2_ plants were obtained by self-pollination of the F_1_ plants.

The leaf morphology of Mizuna, F_1_, and F_2_ generation plants was quantified 4 weeks after sowing. For leaf serration quantification, the serration at the leaf tip was considered a typical serration, and the height of the serrations (SER) was calculated by dividing their width by their height. In each individual, the quantifications were performed with three leaves, and the average values were used for phenotyping. In both 2013 and 2014, we used the F_2_ plants obtained from the same F_1_ plant.

### QTL and RAD-seq analyses

For QTL and RAD-seq analyses, 96 F_2_ individuals from 2013 and 82 F_2_ individuals from 2014 were used, respectively. The molecular markers required for the QTL analysis and the data used for RAD-seq were obtained from our previous study^31^. Genomic DNA used for the QTL analysis was extracted using a DNeasy Plant Mini Kit (Qiagen, Hilden, Germany) according to the manufacturer’s protocol. The QTL analysis was carried out with WinQTL cartographer ver. 2.5^41^ using the composite interval mapping (CIM) method. The CIM analysis was run with forward and backward stepwise regression, a window size of 10 cM, and a step size of 1 cM. A permutation test was conducted (1000 repetitions) to determine the limit of detection (LOD) thresholds (*p* = 0.05).

### RNA-seq analysis

The total RNA was extracted from Mizuna and Mibuna plants grown for 3 weeks at 23 °C using the RNeasy Plant Mini Kit (Qiagen). Shoot apical meristem (SAM) with leaves <0.3 cm in length was sampled. In addition, leaves of lengths 0.4–1, 1–2, and 2–5 cm were sampled. The RNA-seq library was created with TruSeq stranded mRNA (Illumina, San Diego, CA, USA), and sequencing was performed for 76 cycles using NextSeq 500 (Illumina) at a single end. Mapping was performed using TopHat2^42^, and reference *B. rapa* genome sequence (v2.5) was downloaded from the *Brassica* database (BRAD) (http://brassicadb.org/brad/). The number of reads mapped to each gene was counted using HTSeq^43^, and expression variable genes were analyzed for normalization of read count and identification of differentially expressed genes by TCC^44^. A heat map was created from the log FC value obtained by dividing the expression level of Mibuna by Mizuna using the heatmap2 function of the gplots R package (https://cran.r-project.org/web/packages/gplots/index.html).

### Genome sequencing and QTL-seq analysis

DNA for genome sequencing was extracted from the leaves of 3–4 Mizuna and Mibuna individuals using the DNeasy Plant Mini Kit (Qiagen). Genome-seq libraries of each strain were prepared using the Nextera DNA Sample Prep Kit (Illumina). Using Nextseq500 (Illumina), 76 bp paired-end reads were obtained. As reference sequences for QTL-seq and SNP calling, Mizuna genome of *B. rapa* were prepared as follows. Genome-seq reads of Mizuna were mapped to the *B. rapa* genome (v2.5) from BRAD using bowtie2^45^, after which Mizuna genome sequences were generated by converting Mizuna SNPs with Pilon^46^.

For the QTL-seq analysis, two DNA pools were constructed by mixing an equal amount of DNA from 20 F_2_ individuals with low SER scores (SerL; scores of SER = 0.5–1.5) and those with high SER scores (SerH; scores of SER = 2.5–5.0). The QTL-seq library was prepared from these DNA pools using the Nextera DNA Sample Prep Kit, and 76 bp single-end reads were obtained with Nextseq500 (Illumina). The QTL-seq reads from the F_2_ pools and genome-seq reads from Mizuna and Mibuna were mapped to Mizuna genome sequences with bowtie2, and SNP calling was performed using Haplotypecaller in the Genome Analysis Toolkit^47^. The SNPs used to identify QTLs showed assigned genotype quality scores of >40 in Mizuna and Mibuna and >90 in SerH and SerL populations. To identify the candidate genomic loci affecting leaf serrations, 20,084 polymorphic markers were selected to calculate the SNP-index of SerH and SerL based on the genotyping results. The SNP index, that is, the proportion of reads harboring the SNPs that are different from the reference sequence, was detected, and the ΔSNP-index (SerH-SerL) was calculated by subtracting the SNP index of the SerL-pool from that of the SerH-pool. The ΔSNPs-index (Mibuna-Mizuna) and ΔSNPs-index (turnips-Mizuna) were calculated by subtracting the SNP index of Mizuna from that of Mibuna and the SNP index of Mizuna from the of turnip, respectively. Only SNPs of turnips detected between Mizuna and Mibuna were plotted. To facilitate graph visualization, the average ΔSNP-index (SerH-SerL) of the SNPs in each genomic interval was calculated using a sliding window analysis (SWAN) with a 1-Mb window size and 1-kbp increment. To detect the outliers, the interquartile range (IQR) was calculated from all ΔSNP-index scores (SerH-SerL), and the third quartile + 1.5 × IQR was used as the upper limit of the outlier.

### Phylogenetic analysis

A phylogenetic tree was constructed based on amino acid sequences using the neighbor-joining method in MEGA X^48^ (http://www.megasoftware.net/). Reference sequences were obtained from BRAD and GenBank (http://www.ncbi.nlm.nih.gov/). Bootstrap values represent the results of 1000 replicates, and the substitution model used was the Maximum Composite Likelihood Model.

## Supplementary information

Supplementary Figures and tables

## Data Availability

Transcriptomic data and genome sequence data of this study can be found in the DNA Data Bank of Japan (DDBJ) Bioproject data libraries under the following accession numbers: DRA011329 (RNA-seq of Mizuna and Mibuna), DRA011325 (genome sequence of Mizuna, Mibuna, and turnips), and DRA011326 (F_2_ generation between Mizuna and Mibuna for QTL-seq).
