# Supplementary material for: Combination of genetic analysis and ancient literature survey reveals the divergence of traditional Brassica rapa varieties from Kyoto, Japan
Source: Hortic Res. 2021 Jun 1;8:132. doi: 10.1038/s41438-021-00569-0 (PMC8167115; doi:10.1038/s41438-021-00569-0)
Supplement: Supplementary file 1 — Supplementary Figures and tables [file 41438_2021_569_MOESM1_ESM.pdf]

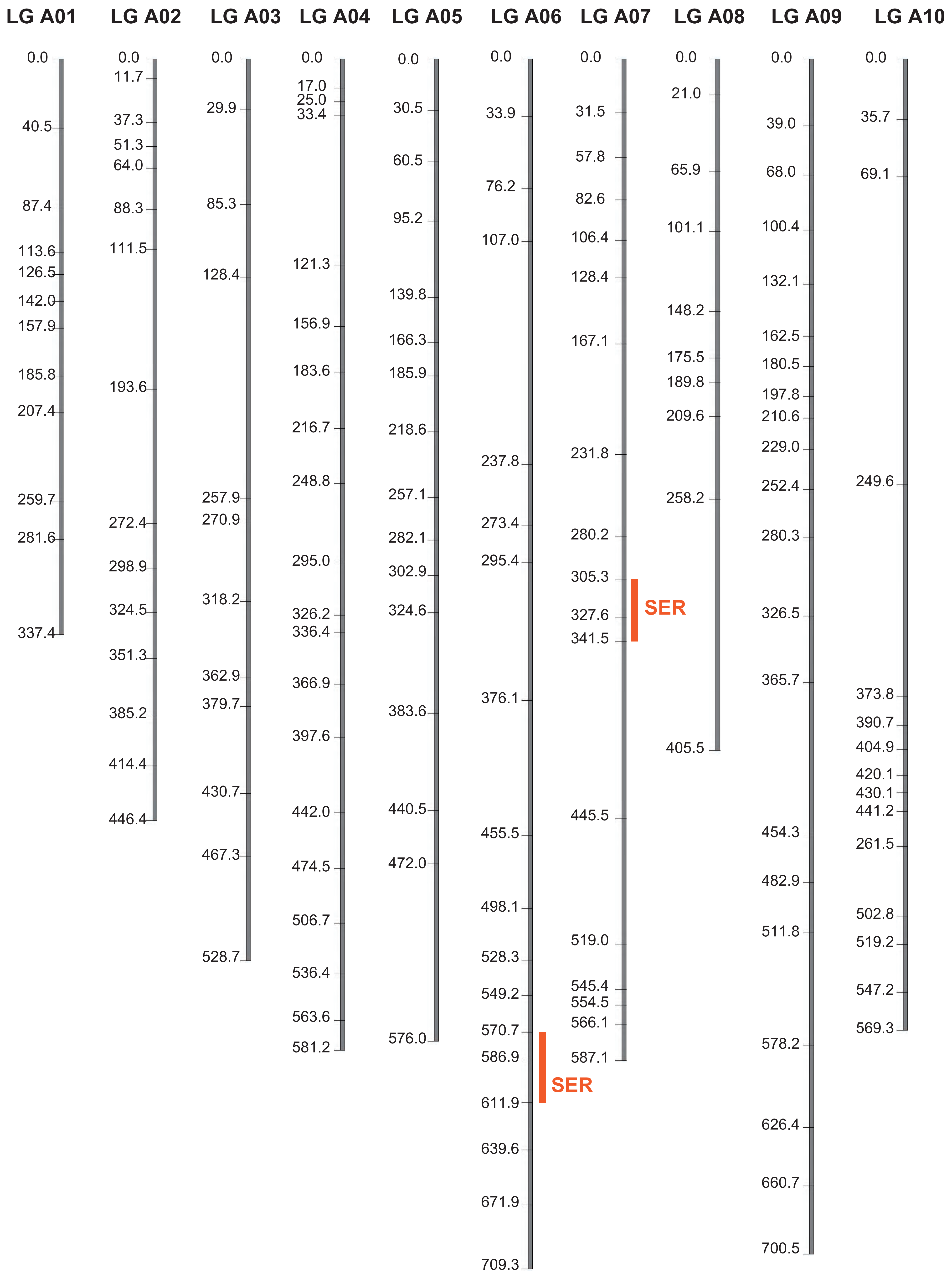


**Supplementary Figure S1 Linkage map and QTLs for serration in Mizuna and Mibuna according to 2014 RAD-seq.** QTL intervals and markers are shown on the right and left of each linkage group, respectively. The names of QTLs are described in Supplementary Table S1.

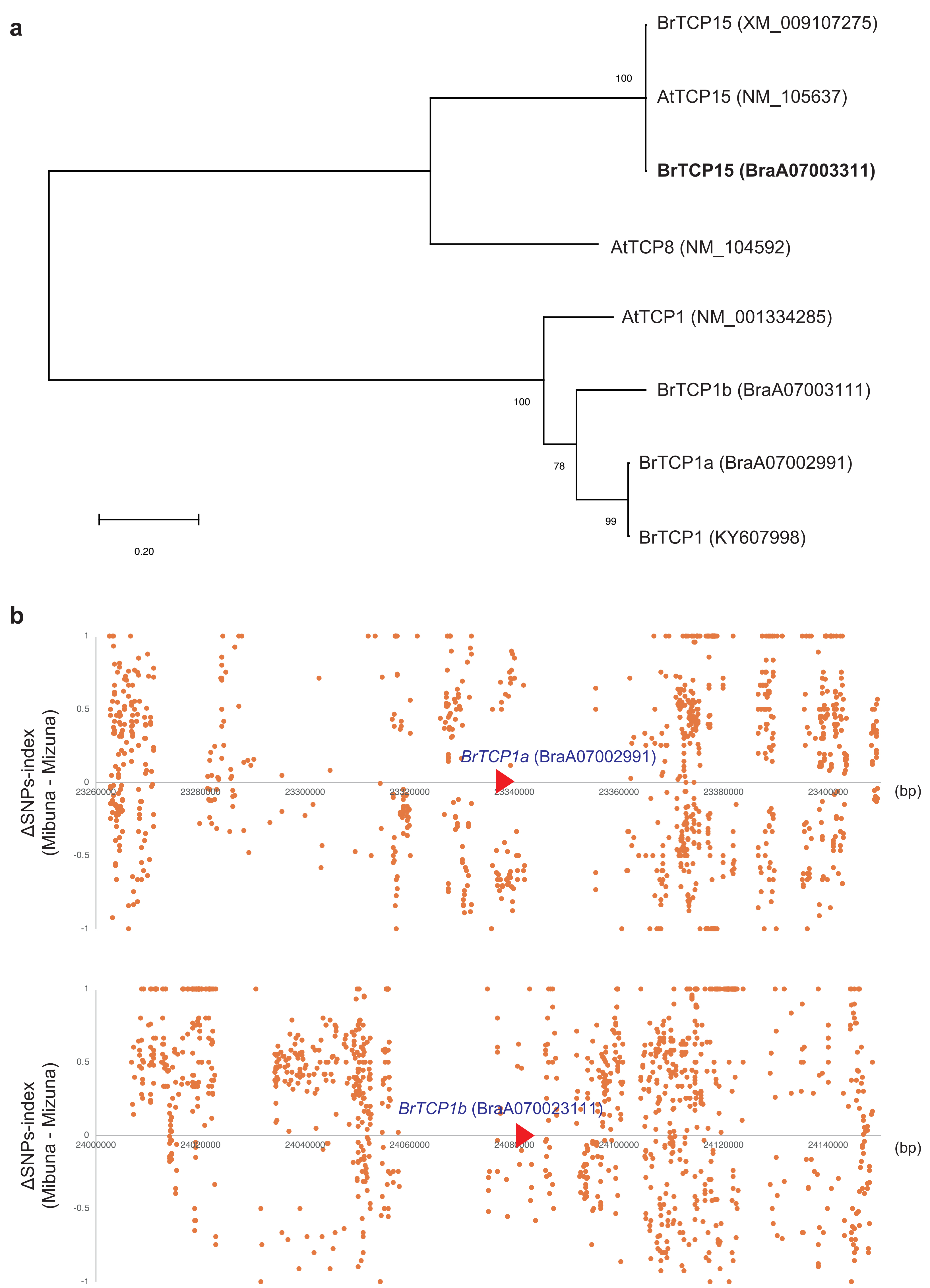


**Supplementary Figure S2 Phylogenetic tree of *BrTCP* orthologs and SNPs around other candidate genes between Mizuna and Mibuna.**

**a** Phylogenetic tree of *BrTCP* orthologs obtained by the neighbor-joining method using MEGA. The bootstrap values > 50% are indicated on branches. **b** The plot of the ΔSNPs-index (Mibuna-Mizuna) around BraA07002991_*BrTCP1a* and BraA07003111_*BrTCP1b*. Red triangles indicate each candidate gene. The vertical axis represents the ΔSNPs-index score, and the horizontal axis represents the position around *BrTCPs* on the chromosome A07 (bp).

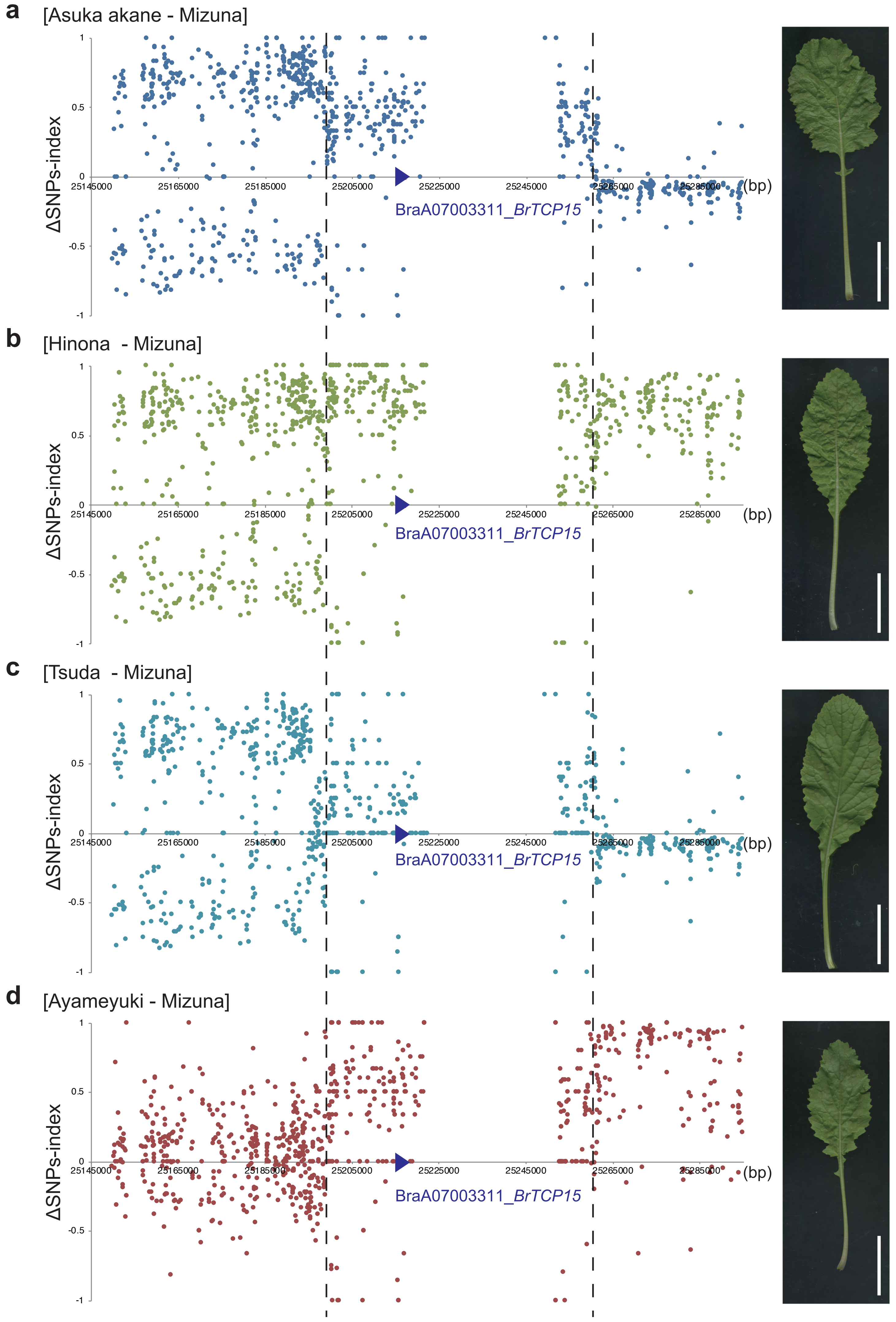


**Supplementary Figure S3 SNPs around *BrTCP15* of other turnips.**
Plot of the ΔSNPs-index (Turnips-Mizuna) around *BrTCP15* of **a** Asuka akane, **b** Hinona, **c** Tsuda, and **d** Ayameyuki. The area between the dashed lines in the plot represents the approximately 61 kbp long locus between 25198 and 25259 kbp. The blue triangle in the center indicates *BrTCP15*. The vertical axis represents the ΔSNPs-index score, and the horizontal axis represents the position around *BrTCP15* on the chromosome A07 (Mb). Scale bar; 3 cm.

Supplementary Table S1 Results of QTL analysis and these genomic locations

| Genotyping | Year | Sample number | Phenotype | LG | Interval* | Location (Mb) | LOD score** | Additive effect | Dominance effect | R2 (%) |
|---|---|---|---|---|---|---|---|---|---|---|
| CAPS markers | 2013 | 96 | Ser | A06 | BraA06001813M-BraA06003805M | 18.9-32.9 | 5.7 | 0.40 | -0.13 | 8.7 |
| | | | | A07 | BraA07003270M-BraA07003440M | 25.0-26.0 | 18.1 | 0.73 | -0.26 | 37.9 |
| | | | | A09 | BraA09003452M-BraA09005859M | 32.0-48.7 | 6.4 | 0.38 | -0.20 | 13.2 |
| RAD-seq | 2014 | 82 | Ser | A06 | 18237-17138 | 19.5-29.7 | 7.6 | 0.46 | -0.41 | 24.7 |
| | | | | A07 | 14298-13693 | 18.3-24.7 | 10.0 | 0.57 | -0.23 | 40.3 |

*Above the LOD threshold

**Scored at the LOD peak

Supplementary Table S2 Genes in QTL on LG A07 and rerative expression levels by RNA-seq

| gene_id | Gene name | logFC SAM Mibuna/Mizuna | logFC 1cm Mibuna/Mizuna | logFC 2cm Mibuna/Mizuna | logFC 5cm Mibuna/Mizuna |
|---|---|---|---|---|---|
| BraA07002927 | | -1.091535417 | 0.606914123 | 0.228021264 | -0.401368952 |
| BraA07002928 | | -0.139702876 | 0.711049054 | -0.33542626 | -0.207808039 |
| BraA07002929 | AtMYB62,BW62B,BW62C,MYB62 | 0 | 0 | 0 | 0 |
| BraA07002930 | | 0 | 0 | 0 | 0 |
| BraA07002931 | | 0 | 0 | 0 | 0 |
| BraA07002932 | | 0 | 0 | 0 | 0 |
| BraA07002933 | | 1.770081854 | -0.646440094 | -0.149703018 | 0.435473312 |
| BraA07002934 | | -0.343600732 | -0.547524366 | -0.321817024 | -0.038832169 |
| BraA07002935 | POLD2 | 0 | 0 | 0 | 0 |
| BraA07002936 | | 0 | 0 | 0 | 0 |
| BraA07002937 | | -0.355244516 | -0.221456803 | -0.297678989 | -0.257895221 |
| BraA07002938 | AtIDD14,IDD14 | -0.259537248 | 0.2318352 | -0.117812688 | -0.034179623 |
| BraA07002939 | IAR1 | -0.555672862 | -0.199024956 | -0.199942663 | -0.327701529 |
| BraA07002940 | | 0.019204281 | -0.778575731 | -0.615554816 | 0.002932149 |
| BraA07002941 | ATMAP7-1,MAP7-1 | -0.327175004 | -0.022975988 | -0.364222208 | -0.257865604 |
| BraA07002942 | ATTPS6,TPS6 | -0.577618165 | -0.286237176 | -0.491340379 | -0.694860378 |
| BraA07002943 | ATHPR1,HPR | 1.915486092 | -1.690100137 | -1.237615101 | -1.068545732 |
| BraA07002944 | ATPIS,ATPIS1,PIS1 | -0.4151152 | -0.165640149 | -0.176760536 | -0.30522881 |
| BraA07002945 | ATLOX2,LOX2 | 1.175962079 | 1.00641702 | 1.868168783 | -1.865147592 |
| BraA07002946 | ATLOX2,LOX2 | 0.259064 | 1.367870508 | 2.930880323 | -1.001026106 |
| BraA07002947 | ATLOX2,LOX2 | 0.769176243 | 0.644358409 | 0.321084178 | -0.494823795 |
| BraA07002948 | ATLOX2,LOX2 | 0.549157435 | 1.763565208 | 2.473813864 | 0.873946536 |
| BraA07002949 | AtENODL22,ENODL22 | 0 | 0 | 0 | 0 |
| BraA07002950 | | 0 | 0 | 0 | 0 |
| BraA07002951 | | -1.471811794 | -1.341745006 | -2.366750715 | -2.000524888 |
| BraA07002952 | | -2.596443857 | 0.574313357 | -0.162335891 | -8.14168E-05 |
| BraA07002953 | | 0.464450641 | 0.039046668 | 0.563659119 | 0.521003666 |
| BraA07002954 | | -0.886782604 | -0.570715301 | -0.620455566 | -0.461447843 |
| BraA07002955 | | -4.264724758 | 2.034792537 | 0.640646345 | -0.421276298 |
| BraA07002956 | | -4.128700734 | 1.166758079 | -0.229824737 | 0.369091754 |
| BraA07002957 | CSK | 0.738759156 | 0.139916166 | 0.032495837 | 0.031989637 |
| BraA07002958 | AtRLP12,RLP12 | 0.519479099 | 0.019746534 | 0.184719871 | 0.234225201 |
| BraA07002959 | AGL53 | 0 | 0 | 0 | 0 |
| BraA07002960 | | 1.141829061 | 0.990440824 | 0.826951748 | 0.759183179 |
| BraA07002961 | | -0.276902267 | 1.756273122 | 0.781661397 | 0.122217686 |
| BraA07002962 | PSBY,YCF32 | 1.972695071 | -0.421246366 | -0.157189906 | -0.02408088 |
| BraA07002963 | ARR11 | -1.385910846 | 0.986514008 | 0.049495509 | -0.420476837 |
| BraA07002964 | | 0.877307553 | -0.701304237 | -0.236795518 | -0.177071708 |
| BraA07002965 | | 0.923138296 | 0.730089511 | 0.909578596 | 0.913071921 |
| BraA07002966 | | 0.024676273 | -0.174953037 | -0.229890199 | 0.209702623 |
| BraA07002967 | | -0.916157748 | 0.771827015 | -0.017969175 | 0.556025625 |
| BraA07002968 | | 0.280873255 | 0.889994383 | 0.379846249 | -0.011567572 |
| BraA07002969 | ORTH2,VIM1 | -6.45607051 | -5.380010996 | 0 | -4.459621729 |
| BraA07002970 | ORTH2,VIM1 | 0.13136871 | 0.418048433 | 0.49620621 | 0.540091947 |
| BraA07002971 | | 0.438563214 | 0.21215495 | -0.201138111 | -0.267644751 |
| BraA07002972 | | 1.539890715 | 1.140214738 | -1.168618107 | 0.629537147 |
| BraA07002973 | | 0 | 0 | 0 | 0 |
| BraA07002974 | ATREV3,REV3 | -0.375559219 | 0.195531809 | 0.102555122 | -0.00402325 |
| BraA07002975 | GCS1,KNF | -0.292372692 | -0.111477398 | 0.119830951 | -0.062178382 |
| BraA07002976 | | -0.18493848 | 3.349189925 | 2.293218145 | 0 |
| BraA07002977 | | 1.769081276 | 0.475840933 | 0.76979212 | 1.169093748 |
| BraA07002978 | | 0.133863236 | 0.512806081 | 0.452920058 | 0.785666913 |
| BraA07002979 | | -0.206707626 | -1.332187781 | 0 | 0 |
| BraA07002980 | | 0 | 0 | 0 | 0 |
| BraA07002981 | | 0.706925656 | 0.868254853 | 0.432271501 | 1.04746146 |
| BraA07002982 | | -0.752746985 | -0.541183842 | -0.790419798 | -0.864260299 |
| BraA07002983 | | 0 | 0 | 0 | 0 |
| BraA07002984 | | 0 | 0 | 0 | 0 |
| BraA07002985 | | 0 | 0 | 0 | 0 |
| BraA07002986 | | 0 | 0 | 10.64678392 | 9.917370889 |
| BraA07002987 | | -0.106135314 | 0.313519764 | 0.225514247 | 0.24304408 |
| BraA07002988 | | 0 | -0.574420696 | 2.172237305 | 0 |
| BraA07002989 | | 0.145838984 | 0.305542199 | 0.366928702 | 0.293958276 |
| BraA07002990 | | -0.628156225 | 0.220031292 | -0.157833166 | -0.3613471 |
| BraA07002991 | TCP1 | 0 | 2.126431457 | 0 | 0 |
| BraA07002992 | | 2.271215797 | 0.77142688 | 0.727490442 | 0.744420851 |
| BraA07002993 | ATHPCAT1,HAC2,HAC2 | 0 | 0 | 0 | 0 |

| | | | | | |
|---|---|---|---|---|---|
| BraA07002994 | | -2.008658665 | -1.812010797 | -2.091554233 | -1.950424161 |
| BraA07002995 | ATHPCAT1,HAC2,HAC2 | 0 | 0 | 0 | 0 |
| BraA07002996 | | -0.130323481 | 0.189168021 | 0.125327028 | -0.012757257 |
| BraA07002997 | GPDL2,MRH5,SHV3 | -1.356247186 | -1.370434519 | -1.211588524 | -0.500807576 |
| BraA07002998 | ATBRXL2,BRX-LIKE2 | 0 | 0 | -0.001859534 | 0 |
| BraA07002999 | | 0 | 0 | 0 | 0 |
| BraA07003000 | ECT3 | 0.280360426 | -0.028857817 | 0.207930493 | 0.009740455 |
| BraA07003001 | ATDSS1(I),DSS1(I) | 0.113118733 | -0.723272973 | -0.433557604 | -0.293522672 |
| BraA07003002 | | -0.026824937 | -0.0639472 | -0.023099803 | -0.265933925 |
| BraA07003003 | | 0.528531212 | -0.075263692 | 0.518529995 | 0.587088099 |
| BraA07003004 | | -5.462907092 | -3.777558927 | 0 | -2.486834312 |
| BraA07003005 | | 0 | -2.273401653 | -3.012603455 | 0 |
| BraA07003006 | 4CL3 | 1.623644742 | 0.016019098 | 0.517066396 | 0.25868061 |
| BraA07003007 | mtACP2 | 0.17844102 | 0.669385141 | 0.849008769 | 0.783460768 |
| BraA07003008 | | 0 | 0 | 0 | 0 |
| BraA07003009 | | 0.9785495 | 0.712378944 | 0.992130481 | 0.619346549 |
| BraA07003010 | | 0.876478114 | 0.579431965 | 0.6154969 | 0.397341012 |
| BraA07003011 | GTB1 | 0.371490173 | 0.481363485 | 0.310640339 | 0.111675891 |
| BraA07003012 | | 0 | 2.136315352 | 0 | 0 |
| BraA07003013 | FAS1,NFB2 | 0.280216413 | 0.410689277 | 0.532058896 | 0.327644001 |
| BraA07003014 | TSF | 0 | 0 | 0 | 0 |
| BraA07003015 | TSF | 0 | 0 | 0 | 0 |
| BraA07003016 | | -0.129211281 | -0.072464102 | -0.138917836 | -0.149113125 |
| BraA07003017 | | 0 | 0 | 0 | 0 |
| BraA07003018 | FRA3 | -0.276519667 | -0.036003975 | -0.159007488 | -0.280745311 |
| BraA07003019 | ATHEX1,HEXO3 | 1.589950788 | 0.990851531 | 1.135993863 | 1.054992179 |
| BraA07003020 | DegP3 | 0 | 0 | 0 | 0 |
| BraA07003021 | UCH2 | 0.29329346 | 0.330523251 | 0.407463456 | 0.208676357 |
| BraA07003022 | | 0.023727759 | -0.281567606 | 0.054844047 | 0.028869617 |
| BraA07003023 | ARF8,ATARF8 | 0.151920257 | 0.555695984 | 0.13666438 | 0.374030197 |
| BraA07003024 | | -1.336504611 | -0.565696427 | -0.706290705 | -0.824077671 |
| BraA07003025 | YSL7 | -1.076671415 | -0.314098297 | -0.340784696 | -0.653953716 |
| BraA07003026 | | -1.244025258 | -0.848436074 | -1.598805202 | -1.619793147 |
| BraA07003027 | BZO1 | 0 | -0.42391998 | 0 | 0 |
| BraA07003028 | | 0.590716298 | 0.971569421 | 0.859075917 | 0.656082918 |
| BraA07003029 | cICDH | 0.026931464 | 0.259927798 | 0.09684397 | -0.096903419 |
| BraA07003030 | | -0.989075603 | -0.240739828 | -0.272158514 | -0.498880036 |
| BraA07003031 | GAD2 | -4.119281411 | -0.852612386 | -1.477089988 | -2.477526768 |
| BraA07003032 | GAD2 | -4.876187588 | -1.553584235 | -1.737804835 | -2.601045908 |
| BraA07003033 | TPX1 | -0.586392432 | 0.990304251 | 0.339205402 | 0.989200005 |
| BraA07003034 | TPX1 | 0.877805119 | 1.649651201 | 0.721917438 | 1.027913298 |
| BraA07003035 | | 0.390147054 | -0.034459821 | 0.480659356 | 0.235631662 |
| BraA07003036 | | 0.076565448 | 0.258049295 | 0.577659088 | 0.42613066 |
| BraA07003037 | | 0 | 0 | 0 | 0 |
| BraA07003038 | | 0.384445323 | 0.165785183 | 0.487192864 | 0.449484544 |
| BraA07003039 | | 0.969234463 | 0.707042385 | 1.233176305 | 1.54067532 |
| BraA07003040 | | 0.314182247 | 0.065086639 | 2.058237178 | -0.035901805 |
| BraA07003041 | | 0 | 0 | 0 | 0 |
| BraA07003042 | | 1.198247159 | -0.158948832 | 0.09899115 | 0.441762897 |
| BraA07003043 | ZFP4 | -5.70009583 | -0.34688136 | -1.276891002 | -0.74247195 |
| BraA07003044 | TMK1 | 0.377456828 | -0.006574235 | -0.295440498 | -0.268739337 |
| BraA07003045 | | 0 | 0 | 0 | 0 |
| BraA07003046 | | 0 | 0 | 0 | 0 |
| BraA07003047 | AtMYB2,MYB2 | -0.81924714 | 0.058323858 | -0.070389057 | -1.285769385 |
| BraA07003048 | | 0 | 0 | 0 | 0 |
| BraA07003049 | | 0 | 0 | 0 | 0 |
| BraA07003050 | | 0.101667791 | 0.106607873 | -0.029435231 | 0.065649074 |
| BraA07003051 | AtETR1,EIN1,ETR,ETR1 | -0.346066673 | 0.322798834 | -0.088412054 | -0.219627233 |
| BraA07003052 | | -1.421952154 | -0.191991431 | 0.629021012 | 3.390256042 |
| BraA07003053 | | 0 | 0 | 0 | 0 |
| BraA07003054 | | 0 | 0 | 0 | 0 |
| BraA07003055 | ATMYB9,MYB9,PAP2 | 0 | 0 | 0 | 0 |
| BraA07003056 | ATMYB9,MYB9,PAP2 | 0 | 0 | 0 | 0 |
| BraA07003057 | | 0 | 0 | 0 | 0 |
| BraA07003058 | | 0 | 0 | 0 | 0 |
| BraA07003059 | CML23 | -0.685433661 | -0.528657158 | -0.671836651 | -0.410914628 |
| BraA07003060 | ACAM-4,CAM4 | -1.349827315 | -0.341492477 | -0.569742687 | -0.63476276 |
| BraA07003061 | | -1.913076288 | 0.55225414 | -1.143568961 | -1.333761598 |
| BraA07003062 | | 1.334583527 | 0.282485872 | 0.451684763 | 0.520008641 |

| | | | | | |
|---|---|---|---|---|---|
| BraA07003063 | RHD6 | 0 | 0 | 0 | 0 |
| BraA07003064 | RHD6 | 0 | 0 | 0 | 0 |
| BraA07003065 | | 2.275277226 | 1.194680897 | 0.875391133 | 0.728503213 |
| BraA07003066 | PMI2 | -2.55024201 | -1.127233983 | -1.996550662 | -2.102144053 |
| BraA07003067 | | -2.304851517 | -1.24490195 | -1.559648744 | -0.669578942 |
| BraA07003068 | | -0.348675054 | -0.6835825 | -0.668742454 | -0.855672467 |
| BraA07003069 | pde194 | 0.728795066 | -0.310556518 | -0.022563766 | 0.302853723 |
| BraA07003070 | | -0.055009512 | -0.064025296 | 0.555400989 | 0.623735137 |
| BraA07003071 | PXMT1 | 0 | -1.489348548 | 1.013031672 | 0.7695577 |
| BraA07003072 | | 0 | 0 | 0 | 0 |
| BraA07003073 | CLPP3,NCLPP3 | 1.125737659 | -0.392093199 | 0.064330206 | 0.239573076 |
| BraA07003074 | AR41 | 0.11733744 | 0.011434871 | 0.218611251 | 0.166194058 |
| BraA07003075 | | 0 | 0 | 0 | 0 |
| BraA07003076 | ARK3,RK3 | 0 | 0 | 0 | 0 |
| BraA07003077 | | 0 | 0 | 0 | 0 |
| BraA07003078 | | 0 | 0 | 0 | 0 |
| BraA07003079 | | 1.604467362 | 0.89432967 | 0.346518835 | 1.726270841 |
| BraA07003080 | | 0 | 0 | 0 | 0 |
| BraA07003081 | PXMT1 | 0 | 0 | 0 | 0 |
| BraA07003082 | | 0 | 0 | 0 | 0 |
| BraA07003083 | PXMT1 | 0 | 0 | 0 | 0 |
| BraA07003084 | AtLIG6,LIG6 | -1.978340404 | -1.616150361 | -1.657079219 | -1.881860758 |
| BraA07003085 | ASF1A,AtSP7,SGA2,SP7 | 0.141324696 | 0.161221162 | 0.361026127 | 0.294400525 |
| BraA07003086 | | -0.114712618 | -0.199090461 | -0.05095504 | -0.125998503 |
| BraA07003087 | | -3.969236905 | -4.453683318 | -5.220977041 | -4.508390234 |
| BraA07003088 | | -1.718773917 | -0.482505979 | -0.367275239 | -0.271577016 |
| BraA07003089 | | -1.712144334 | -0.324311975 | -0.797308256 | -0.121752353 |
| BraA07003090 | | -2.82108998 | -3.412294854 | -2.872343526 | -2.320273775 |
| BraA07003091 | | -4.072204863 | -0.946905393 | -0.94415619 | -1.717143146 |
| BraA07003092 | | -1.032084829 | -2.013052697 | -1.899313328 | -1.399954509 |
| BraA07003093 | | 0 | 0 | 0 | 0 |
| BraA07003094 | | -2.121219143 | -1.849176411 | -2.485376581 | -1.775223443 |
| BraA07003095 | | -2.072985272 | -1.764076469 | -2.068683699 | -2.24916127 |
| BraA07003096 | | 0 | -2.946712792 | 0 | 0 |
| BraA07003097 | | 0.23731668 | -0.352292014 | -0.515182264 | -0.122883579 |
| BraA07003098 | | 0 | 0 | 0 | 0 |
| BraA07003099 | | 0 | 0 | 0.18197959 | 0 |
| BraA07003100 | | -0.196479526 | 0.432375412 | -0.798050474 | -1.711405029 |
| BraA07003101 | ZFP6 | -3.660242414 | 0.458934413 | -0.315984052 | -0.862475681 |
| BraA07003102 | ATBX3,ATBXL3,BX3,BXL3,XYL3 | 0 | -0.538833457 | 0 | 0 |
| BraA07003103 | | -1.614883655 | -2.848261043 | -2.564414255 | -2.247323207 |
| BraA07003104 | | -1.293774746 | -0.500414248 | -0.788587993 | 0.092641603 |
| BraA07003105 | | 0 | 0 | 0 | 0 |
| BraA07003106 | | 0 | 0 | 0 | 0 |
| BraA07003107 | SWEETIE | 0.166734157 | 0.227674159 | -0.117009563 | -0.227503801 |
| BraA07003108 | | -0.403514516 | -0.264229825 | -0.069344097 | -0.296029815 |
| BraA07003109 | | -0.368688813 | -0.361231031 | -0.126698191 | -0.137398065 |
| BraA07003110 | | -0.352770519 | -0.613382153 | -0.487447122 | -0.270149074 |
| BraA07003111 | TCP1 | -2.191849095 | 2.036304258 | 0 | 0 |
| BraA07003112 | | 0.721522095 | 0.505487097 | 0.638691788 | 0.567059451 |
| BraA07003113 | | 0 | 0 | 0 | 0 |
| BraA07003114 | | 0.838523674 | -0.485818085 | -0.10159041 | -0.3135548 |
| BraA07003115 | | -0.029595068 | 3.298602058 | 0.320746792 | -1.220475433 |
| BraA07003116 | | -0.477118874 | -0.024457688 | 0.027461543 | 0.043801136 |
| BraA07003117 | | 0.322003164 | 0.950577551 | 0.952442795 | 1.154135536 |
| BraA07003118 | | -0.922090953 | -0.823433086 | -0.645882084 | -0.554702031 |
| BraA07003119 | | 0 | 0 | 0 | 0 |
| BraA07003120 | | -0.433053056 | -0.008677928 | -0.132675209 | -0.091079817 |
| BraA07003121 | | 0.689610253 | 0.020416234 | 0.031369462 | 0.033784137 |
| BraA07003122 | ASY1,ATASY1 | -0.490827432 | 0.917899436 | 0.857565576 | 0.32012361 |
| BraA07003123 | | 0.016055429 | 0.163259496 | -0.334983126 | -0.239770927 |
| BraA07003124 | | 0 | 0 | 0 | 0 |
| BraA07003125 | | 0.539478203 | -0.38564138 | -0.077153168 | 0.156563535 |
| BraA07003126 | emb1688 | -0.106601431 | -0.671847011 | -0.403230076 | -0.651428827 |
| BraA07003127 | | -0.753666307 | 0.39047499 | 0.31529017 | -0.131631219 |
| BraA07003128 | | -0.606177004 | 0 | -0.482459396 | 0.347169521 |
| BraA07003129 | | 0.770260948 | 0.526103068 | 0.738139955 | 0.317701049 |
| BraA07003130 | | 0 | 0 | 0 | 0 |
| BraA07003131 | | 0.681137083 | -0.051102229 | -0.054198013 | 0.039263903 |

| | | | | | |
|---|---|---|---|---|---|
| BraA07003132 | | 0.546067878 | 0.129162446 | 0.69887721 | 0.527976805 |
| BraA07003133 | | 0 | 0 | 0 | 0 |
| BraA07003134 | | 0 | 0 | 0 | 0 |
| BraA07003135 | | 0 | 0 | -0.061333817 | -0.559903754 |
| BraA07003136 | | -0.50497837 | -0.103243735 | -0.103139629 | -0.843374725 |
| BraA07003137 | | 0.272839068 | 0.185537063 | 0.18146074 | -0.000192902 |
| BraA07003138 | | -0.342266655 | -0.413115897 | -0.433760785 | 0.4716235 |
| BraA07003139 | | -0.394135448 | 1.96253805 | 0.722442548 | 1.998850006 |
| BraA07003140 | | 0.975822216 | 0.55297656 | 0.656013873 | 0.37399705 |
| BraA07003141 | | 0.906656685 | -0.925173204 | -0.480137796 | -0.456697509 |
| BraA07003142 | | 0 | 0 | 0 | 0 |
| BraA07003143 | | -0.576410429 | -0.623701297 | -0.414911928 | -0.613260032 |
| BraA07003144 | | 0.766247508 | -0.321612382 | -0.07262735 | 0.027091202 |
| BraA07003145 | ARR11 | -3.559579498 | -0.979471333 | -1.555205338 | -0.785845951 |
| BraA07003146 | | -0.473180764 | 2.590255939 | 0.661011386 | -1.588614749 |
| BraA07003147 | FTSH5,VAR1 | 0.64707027 | -0.626595984 | -0.438232516 | -0.571789293 |
| BraA07003148 | | -0.276109213 | 0.74989458 | 0.442600061 | 0.479427065 |
| BraA07003149 | | 0.203047654 | -1.35761432 | -0.976239484 | -0.073613273 |
| BraA07003150 | ATKCR1,KCR1,YBR159 | 0.115049794 | -0.282551395 | -0.422767429 | -0.581708991 |
| BraA07003151 | PSBY,YCF32 | 1.202199824 | -0.480011996 | -0.041283005 | 0.103669153 |
| BraA07003152 | | -1.174001764 | -0.143938905 | -0.667084633 | -0.294509412 |
| BraA07003153 | | -0.170875731 | 1.221599041 | -0.083942543 | -0.570856486 |
| BraA07003154 | TEL2 | -4.591365576 | 0 | 0 | 0 |
| BraA07003155 | | -0.108218654 | -0.242235148 | -0.011738859 | 0.325127651 |
| BraA07003156 | SDG17,SUVH7 | 0 | 0 | 0 | 0 |
| BraA07003157 | | -1.264480683 | 0.900670372 | 0.871951109 | -0.206406126 |
| BraA07003158 | | -0.540405767 | -0.647469922 | -0.744542112 | -0.794326864 |
| BraA07003159 | SUFE2 | 0 | 0 | 0 | 0 |
| BraA07003160 | | 0 | 0 | 0 | 0 |
| BraA07003161 | ATFXG1,FXG1 | 0.600538059 | 0.138137801 | 0.319051001 | 0.401119685 |
| BraA07003162 | | 0 | 0 | 0 | 0 |
| BraA07003163 | | 0 | 0 | 0 | -1.478181462 |
| BraA07003164 | | -8.857167391 | -3.649734067 | -5.092559535 | -6.499303646 |
| BraA07003165 | | -1.167655399 | -0.083868817 | -0.727708257 | -1.12160468 |
| BraA07003166 | | -0.462501575 | -0.053816905 | -0.207934148 | -0.158539183 |
| BraA07003167 | | -0.646535676 | -0.662403824 | -0.809062877 | -0.71540003 |
| BraA07003168 | ATNAP3,AtSTAR1,NAP3 | -1.804420332 | -2.605346982 | -2.742514316 | -2.992816748 |
| BraA07003169 | AT-HSFA8,HSFA8 | -0.796673111 | -0.829559345 | -1.141183114 | -1.080570009 |
| BraA07003170 | CCOAMT | 0 | 0 | 0 | 0 |
| BraA07003171 | ATPIS,ATPIS1,PIS1 | 0.563140793 | 0.613618495 | 0.802769072 | 0.769500912 |
| BraA07003172 | ATHPR1,HPR | 0.286677019 | -1.795890701 | -1.637129839 | -1.55951582 |
| BraA07003173 | | -0.283905955 | -0.194204067 | -0.080358451 | 0.346500352 |
| BraA07003174 | | 0 | 0 | -2.888853218 | 0 |
| BraA07003175 | | 0 | 0 | 0 | 0 |
| BraA07003176 | ATMAP7-1,MAP7-1 | -0.103327428 | -0.166388508 | -0.197610363 | -0.29314528 |
| BraA07003177 | | -0.247315592 | -0.321957845 | -0.280669068 | -0.136406248 |
| BraA07003178 | IAR1 | 0.557128821 | 0.4471724 | 0.40432314 | 0.382808768 |
| BraA07003179 | | -0.238244991 | -0.077290043 | -0.841045885 | -1.069100784 |
| BraA07003180 | AtIDD14,IDD14 | -0.146156575 | 0.886443793 | -0.192201194 | -0.33073938 |
| BraA07003181 | | 0.064193871 | -0.043118412 | 0.002237484 | 0.01376066 |
| BraA07003182 | ATWRKY9,WRKY9 | -3.411401772 | 0 | 0.456322919 | 0.816254813 |
| BraA07003183 | | 0.3798884 | 0.553977044 | 0.458478196 | 0.578693338 |
| BraA07003184 | | -0.513685654 | 0.160367134 | -1.116735474 | -1.28855247 |
| BraA07003185 | | -0.027340154 | 0.575227518 | -0.736723604 | 0.777010437 |
| BraA07003186 | APRR6,PRR6 | 0.530257968 | 0 | -1.803979288 | 0 |
| BraA07003187 | | -0.266287842 | -0.238153527 | -0.580039211 | -0.640142318 |
| BraA07003188 | | -2.617471384 | -1.730034 | -2.050319939 | -3.740453628 |
| BraA07003189 | | -0.946256663 | -0.166381751 | -0.198336328 | -0.38360649 |
| BraA07003190 | | 0.901069657 | -0.632744688 | 0.043356756 | 0.009895418 |
| BraA07003191 | ENDO 2 | 0.615272522 | 0.329149855 | 2.282231805 | 0.334353911 |
| BraA07003192 | | -0.041157145 | 0.231120413 | 0.051227058 | -0.025760798 |
| BraA07003193 | | 1.643705781 | 0.333855533 | 0.255686305 | -0.170217714 |
| BraA07003194 | AtMYB62,BW62B,BW62C,MYB62 | 0 | 0 | 0 | 0 |
| BraA07003195 | | -0.908764311 | 0.910925686 | -0.115502767 | -0.003522951 |
| BraA07003196 | | -0.029665461 | 1.322794919 | -0.621264009 | 1.138358455 |
| BraA07003197 | | -1.750240082 | 0.507319654 | -0.272964227 | 0.200991253 |
| BraA07003198 | ARG1 | 0.226297207 | 0.116809523 | 0.297812374 | 0.124151532 |
| BraA07003199 | | 0 | 0 | 0 | 0 |
| BraA07003200 | | -0.888018335 | 0.402024207 | 0.25168643 | -0.626497889 |

| | | | | | |
|---|---|---|---|---|---|
| BraA07003201 | APRR6,PRR6 | 0 | 0 | 0 | 0 |
| BraA07003202 | ATNDT2,NDT2 | -0.841964001 | 0.905721413 | 1.227593024 | 0.106781039 |
| BraA07003203 | | -0.646998654 | 0.307351535 | 0.320777473 | 0.235015154 |
| BraA07003204 | | -0.607549493 | -0.216186569 | -0.564066892 | -1.388834192 |
| BraA07003205 | | 2.070128142 | 1.558192925 | 1.682472288 | 2.368695461 |
| BraA07003206 | JAG | 0.781778734 | 1.209048251 | 2.33171954 | 3.276204485 |
| BraA07003207 | | 0.279252156 | -0.001167572 | -0.149017975 | -0.377926439 |
| BraA07003208 | | 0.701017001 | -1.392980566 | -0.519344363 | -1.461544316 |
| BraA07003209 | LBD42 | 0 | 0 | 0 | 0 |
| BraA07003210 | | 0 | 0 | 0 | 0 |
| BraA07003211 | | 0.046948651 | -0.094282185 | 0.150127712 | 0.550409967 |
| BraA07003212 | ATXYL1,TRG1,XYL1 | 0.182109838 | -0.489149785 | -0.069898792 | -0.317198759 |
| BraA07003213 | | -3.234821795 | -4.061532227 | -2.848453914 | -3.207279265 |
| BraA07003214 | | 1.115910634 | 0.166681158 | 0.474008319 | 0.762617148 |
| BraA07003215 | | -0.079087564 | 0.658794407 | 1.350171739 | 0.513374084 |
| BraA07003216 | | 0.187810159 | 0.334307225 | 0.237383542 | 0.437303539 |
| BraA07003217 | | 1.794528251 | -1.052974936 | -0.191640294 | -0.042408428 |
| BraA07003218 | | -0.232827834 | -1.367230497 | -0.329291852 | 0.190287738 |
| BraA07003219 | PCR11 | 0 | 0 | 0 | 0 |
| BraA07003220 | | 0 | -1.42391998 | 0 | -1.927492654 |
| BraA07003221 | | 0 | 0 | 0 | 0 |
| BraA07003222 | PAN | -0.074382985 | 0.522508487 | 1.310262797 | 1.609015287 |
| BraA07003223 | | 0.206095305 | -0.150111356 | 0.358316328 | 0.161456081 |
| BraA07003224 | | -0.608750193 | 0.027260711 | 0.23530095 | -0.755286908 |
| BraA07003225 | | 5.151517626 | 4.475619967 | 5.712441533 | 6.639624576 |
| BraA07003226 | | 0.163433312 | 1.119597536 | 0.776742597 | -0.065536146 |
| BraA07003227 | | -0.29351857 | 0.154589791 | 0.048917818 | 0.212897579 |
| BraA07003228 | | 0.195230074 | -0.26736661 | -0.376092448 | -0.487795904 |
| BraA07003229 | | -1.09333706 | 0.680300954 | -0.018352478 | 0.134989243 |
| BraA07003230 | | -0.177760049 | -0.901689506 | -0.543233267 | -0.125239696 |
| BraA07003231 | PHO1;H1 | -1.991603632 | 1.228106878 | 0.884435511 | 0.58096508 |
| BraA07003232 | IDA | 0 | 0 | 0 | 0 |
| BraA07003233 | | 1.810105086 | -0.211716086 | -0.012783912 | 0.180415181 |
| BraA07003234 | CLE12 | -1.222949991 | 2.227191343 | 0.045536864 | -0.265484141 |
| BraA07003235 | | 0.431547624 | 0.407788788 | 0.113051319 | 0.075466902 |
| BraA07003236 | | -0.813741332 | -0.300842889 | -0.281446208 | -0.329717747 |
| BraA07003237 | STN7 | 0.286935283 | -0.646999327 | -0.636139678 | -0.676317177 |
| BraA07003238 | SSP | 0 | 0 | 0 | 0 |
| BraA07003239 | | 0 | 0 | 0 | 0 |
| BraA07003240 | | 0 | 0 | 0 | 0 |
| BraA07003241 | ATSOFL2,SOFL2 | 0.865388528 | -1.270704943 | 0.739921106 | 1.206313191 |
| BraA07003242 | | 0 | 0 | 0 | 5.920535764 |
| BraA07003243 | | 0 | 0 | 0 | 0 |
| BraA07003244 | | 0 | 0.526199824 | 0 | -0.619569347 |
| BraA07003245 | WIT2 | 0 | 0 | 0 | 0 |
| BraA07003246 | WIT2 | -0.213790744 | -0.097187884 | -0.134068816 | -0.684128605 |
| BraA07003247 | | -0.871664319 | -0.79255156 | -0.863058117 | -1.111722875 |
| BraA07003248 | | -0.280814949 | -0.425472901 | -1.363849225 | -1.143031686 |
| BraA07003249 | | -7.254708438 | -2.604451105 | -4.046235414 | -4.440188406 |
| BraA07003250 | | 0 | 0 | 0 | 0 |
| BraA07003251 | | 0 | 0 | 0 | 0 |
| BraA07003252 | MGP3 | 0.157480564 | 0.087961868 | 0.171250507 | -0.225642856 |
| BraA07003253 | BIM2 | 0.588313291 | 0.281623782 | 0.521239362 | 0.425870673 |
| BraA07003254 | | -1.18973137 | -0.725444923 | -0.517272159 | -0.742924282 |
| BraA07003255 | ACR4 | 0.215754822 | -0.568334974 | -0.211955033 | -0.411332878 |
| BraA07003256 | | -0.701874927 | 0.594517562 | 0.467287397 | 0.676557703 |
| BraA07003257 | | -0.596693227 | -0.469945774 | -0.56443393 | -0.638121543 |
| BraA07003258 | | 1.149146508 | -0.181508951 | -0.164674497 | 0.011034641 |
| BraA07003259 | | 0 | 0 | 0 | 1.621794751 |
| BraA07003260 | AGL7,AP1 | 0 | 0 | 0 | 0 |
| BraA07003261 | | 1.446087626 | 0.146789412 | 0.353031631 | 0.198496224 |
| BraA07003262 | | -0.362646297 | -0.003888223 | 0.018522506 | 0.165098762 |
| BraA07003263 | CRC | 0.058658791 | 1.421466192 | 3.821212459 | 1.856416405 |
| BraA07003264 | | 0 | 0 | 0 | 0 |
| BraA07003265 | FLN2 | 1.117309461 | -0.610860369 | -0.026796066 | 0.12456844 |
| BraA07003266 | SIK1 | -0.236105897 | -0.124680406 | -0.221893266 | -0.256115213 |
| BraA07003267 | | 0.101224521 | -0.046365269 | 0.341607931 | 0.142695294 |
| BraA07003268 | AFP1 | 0 | 0 | 0 | 0 |
| BraA07003269 | RPK1 | 0 | 0 | 0 | 0 |

| | | | | | |
|---|---|---|---|---|---|
| BraA07003270 | | -0.478588084 | -0.014686758 | 0.08456409 | 0.169813267 |
| BraA07003271 | ATWRKY57,WRKY57 | 0.304226574 | 2.877979705 | 4.016458239 | 1.453062995 |
| BraA07003272 | CLE1 | 1.153977012 | -2.245215962 | -0.603464198 | -0.641608757 |
| BraA07003273 | | -1.335686662 | -0.28816119 | -1.4843648 | -1.228983528 |
| BraA07003274 | | -0.491833762 | 0.154550456 | 0.20079489 | 0.362862883 |
| BraA07003275 | | -0.126503717 | -0.106777952 | -0.0747777 | 0.013979095 |
| BraA07003276 | | -0.048753505 | -0.425951041 | -0.542043817 | -0.15301405 |
| BraA07003277 | cm-3,CM3 | 0.561872405 | 0.525210384 | 1.62238405 | 1.080576127 |
| BraA07003278 | | 0.426176501 | -0.255211733 | -0.061692218 | -0.038569564 |
| BraA07003279 | ARC12,ATMINE1,MINE1 | 0.861108101 | -0.312368389 | 0.038201064 | 0.203516819 |
| BraA07003280 | ATELF5A-3,ELF5A-3 | -2.158345668 | -0.214082764 | -0.445615343 | -0.54622332 |
| BraA07003281 | | 0 | 0 | 0 | 0 |
| BraA07003282 | | 0 | 0 | 0 | 0 |
| BraA07003283 | | 0.142282274 | -0.306464329 | -0.015640157 | 0.302416381 |
| BraA07003284 | | 0.776233525 | 0.048365518 | -0.028050007 | 0.292563107 |
| BraA07003285 | | -0.033684793 | -0.003647734 | 0.140771938 | 0.073330474 |
| BraA07003286 | | -1.843239296 | 0 | 0 | 0 |
| BraA07003287 | | 0.303592499 | 0.55609278 | 1.157457599 | 0.970073309 |
| BraA07003288 | ANAC29,ATNAP,NAP | 1.626892887 | 0 | 2.821134905 | 2.55465453 |
| BraA07003289 | CYP74B1 | 0 | 0 | 0 | 0 |
| BraA07003290 | | -0.201836665 | 0.042750973 | 0.152713652 | 0.206129814 |
| BraA07003291 | DRM1 | -1.749147321 | -0.204995225 | -0.232697666 | -0.137996273 |
| BraA07003292 | | -1.267702816 | -1.294940222 | -1.328368631 | -0.992533126 |
| BraA07003293 | | 0 | 0 | 0 | 0 |
| BraA07003294 | AT-EXP1,ATEXP1,ATEXPA1,ATHE | -0.480557263 | -1.049806624 | -0.209708984 | 0.052770864 |
| BraA07003295 | AGL94 | -0.169809027 | -0.324840867 | -2.137171571 | -1.412656728 |
| BraA07003296 | ATMYB15,LOF2,MYB15 | -1.57559942 | 0.695087379 | -0.2584281 | 0 |
| BraA07003297 | CARA | 0.876566586 | 0.094042285 | 0.447446369 | 0.306962324 |
| BraA07003298 | | 1.748483978 | 1.694924223 | 3.155127278 | 1.155065828 |
| BraA07003299 | | -0.328833076 | 0.76500836 | -0.035685826 | -0.166040919 |
| BraA07003300 | CLE45 | -1.24948885 | 0.122585654 | 0.236069426 | -0.832757995 |
| BraA07003301 | | 0 | 0 | 0 | 0 |
| BraA07003302 | | 0 | 0 | 0 | 0 |
| BraA07003303 | ATHB29,ZFHD1 | -1.996368338 | -0.0771219 | -0.2908072 | -0.508143673 |
| BraA07003304 | RPL34 | 0.810868157 | -0.027235497 | 0.100142234 | 0.336410119 |
| BraA07003305 | | 0 | 0 | 0 | 0 |
| BraA07003306 | | -2.565220526 | -1.803742376 | -2.508243348 | -2.221934294 |
| BraA07003307 | ANAC9,FEZ | 0 | 0 | 0 | 0 |
| BraA07003308 | SBH1 | -0.058183239 | 0.428055288 | 0.268287617 | 0.107212295 |
| BraA07003309 | | 0 | 0 | 0 | 0 |
| BraA07003310 | | -1.358515035 | -1.365834825 | -1.636301106 | -2.125156152 |
| BraA07003311 | TCP15 | -0.654302922 | -1.836027063 | -2.542691743 | -2.54908589 |
| BraA07003312 | ATHVA22C,HVA22C | -0.507305723 | -0.11053485 | -0.315242345 | -0.58336982 |
| BraA07003313 | ATERF-8,ATERF8,ERF8 | 0 | 0 | 0 | 0 |
| BraA07003314 | HEMB1 | 1.06383972 | -0.427932502 | 0.080928944 | 0.015730544 |
| BraA07003315 | RPL1C,SAG24 | 0.709543927 | -0.068050411 | 0.217396083 | 0.479313224 |
| BraA07003316 | ATCOX19-2,COX19-2 | 0.444095017 | -0.070678887 | 0.146726118 | 0.146694015 |
| BraA07003317 | | -0.445425963 | -0.52038193 | -0.436783604 | -1.508031714 |
| BraA07003318 | ATHB13 | 0.212048087 | 0.885341502 | -0.136367373 | 0.058138077 |
| BraA07003319 | | -3.959236104 | -0.8790518 | -0.425097712 | -1.044290267 |
| BraA07003320 | ATUBC2,UBC2 | -0.218548852 | 0.085066184 | 0.001534735 | -0.105145336 |
| BraA07003321 | | 2.007682573 | 0.951876892 | 0.955252912 | 0.97340173 |
| BraA07003322 | | 0 | 0 | 0 | 0 |
| BraA07003323 | AMY3,ATAMY3 | 0.058761421 | -0.04900579 | -0.105910593 | -0.418518993 |
| BraA07003324 | | -0.407897429 | -0.227131843 | -0.170880626 | -0.390399819 |
| BraA07003325 | | 0 | 0 | 0 | 0 |
| BraA07003326 | | 0 | 0 | 0 | 0 |
| BraA07003327 | NRT1.7 | -0.237213693 | 2.079029788 | 0.914893459 | -0.52974533 |
| BraA07003328 | ATH8,TH8 | -4.147111883 | 0.011302919 | 0.014825807 | 0.326024446 |
| BraA07003329 | | 0.659986792 | -0.62138766 | -0.145763005 | -0.062667558 |
| BraA07003330 | | 0 | 0 | 0 | 0 |
| BraA07003331 | | 0.139861977 | -1.500601889 | -1.584068644 | -1.543773037 |
| BraA07003332 | | -1.98145042 | -1.518990293 | -2.043390913 | -1.136075401 |
| BraA07003333 | ATGSTU12,GSTU12 | -0.040898739 | 0.719152717 | -0.08407556 | -0.062795447 |
| BraA07003334 | ATGSTU12,GSTU12 | 0.920318158 | -1.753822378 | -2.41117852 | -1.458809745 |
| BraA07003335 | iqd9 | -1.533883953 | -1.723365999 | -1.566756738 | -1.392266224 |
| BraA07003336 | ATGSTU11,GSTU11 | 0.225231962 | -2.346814567 | -2.47978451 | -1.343087606 |
| BraA07003337 | SHW1 | 0.502770241 | -0.388648821 | -0.307242829 | 0.050609887 |
| BraA07003338 | | -0.122802444 | -0.081306129 | 0.033791375 | -0.063511765 |

| | | | | | |
|---|---|---|---|---|---|
| BraA07003339 | PP2A | -0.547953833 | -0.456162587 | -0.615267993 | -0.491998114 |
| BraA07003340 | CLE26 | -0.346482727 | -0.1315724 | -0.447969655 | 0.204960536 |
| BraA07003341 | | -0.29312544 | -0.271966349 | -0.456012864 | -0.392722506 |
| BraA07003342 | | -4.68747763 | 1.40066095 | -2.402894539 | -0.91327674 |
| BraA07003343 | | 0 | 0 | 0 | 0 |
| BraA07003344 | | 0 | 0 | 0 | 0 |
| BraA07003345 | SNL4 | -0.020553709 | 0.113724112 | -0.127337828 | -0.206728287 |
| BraA07003346 | EMB25,ISE2,PDE317 | 0.907634538 | -0.347235699 | -0.104164723 | -0.108798109 |
| BraA07003347 | | 0 | 0 | 0 | 0 |
| BraA07003348 | IAR4 | -0.816149853 | -0.143736224 | 0.092144142 | -0.362507641 |
| BraA07003349 | GATL9,LGT8 | 0.854628935 | 0.482870426 | 0.031090353 | 0.391683258 |
| BraA07003350 | | -0.139473334 | 0.329438158 | 0.226560648 | 0.268138311 |
| BraA07003351 | | 0 | 0 | 0 | 0 |
| BraA07003352 | | 0 | 0 | 0 | 0 |
| BraA07003353 | | 1.333544553 | 2.720344496 | -0.054274092 | 1.008348803 |
| BraA07003354 | ATFH8,FH8 | -0.149355127 | -0.016228135 | -1.373444795 | 0.107942406 |
| BraA07003355 | | 0.52759028 | 0.492108419 | 0.42476626 | 0.320268323 |
| BraA07003356 | | -0.257591825 | -0.136182335 | -0.4004396 | -0.520265127 |
| BraA07003357 | | 0 | 0 | 0 | 0 |
| BraA07003358 | | 0 | 0 | 0 | 0 |
| BraA07003359 | MMP | 1.890945807 | -0.709900132 | -0.718137346 | -0.400273698 |
| BraA07003360 | ATCYCD1;1,CYCD1;1 | 1.404705321 | 0.521268122 | 0.572906 | 0.99951361 |
| BraA07003361 | TBL27 | -1.124842443 | 0.123152028 | 0.015940256 | 0.680445153 |
| BraA07003362 | | -8.202331942 | 0 | -0.7177701 | 0.122503092 |
| BraA07003363 | | -1.144343838 | -0.28506005 | -0.403188966 | -0.242489162 |
| BraA07003364 | | 0 | 0 | 0 | 0 |
| BraA07003365 | | -1.149715116 | -2.375615449 | -2.677885534 | -3.191679307 |
| BraA07003366 | KUP6 | -1.280184071 | -1.267595861 | -1.160563106 | -0.959194093 |
| BraA07003367 | ENT1,ENT1AT | 0 | 0 | 0 | 0 |
| BraA07003368 | PG2 | -0.095921898 | 0.179186264 | -0.131347622 | -0.346596199 |
| BraA07003369 | | 0 | -0.458531044 | 0 | 0 |
| BraA07003370 | ATBCA4,BCA4,CA4 | -0.529754467 | -1.634485623 | -0.021364591 | -0.06287452 |
| BraA07003371 | | 0 | 0 | 0 | 0 |
| BraA07003372 | | -0.718067587 | -0.11378968 | -0.695551779 | -1.563068126 |
| BraA07003373 | | 0.143308657 | -0.406552625 | -1.026449138 | -1.35389833 |
| BraA07003374 | SRO3 | 0 | 0 | 0 | 0 |
| BraA07003375 | RHS1 | -5.43168549 | -4.870532643 | -6.301740444 | 0 |
| BraA07003376 | | -0.022514301 | -0.148148246 | 0.35022392 | 0.292933174 |
| BraA07003377 | | -0.444861763 | -0.418407078 | -0.444959138 | -0.14059525 |
| BraA07003378 | ARF1,ATARF,ATARF1,ATARFA1A | -0.681306148 | -0.366858592 | -0.413044848 | -0.572166967 |
| BraA07003379 | | -0.204885915 | -0.299199122 | -0.259231984 | 0.058831291 |
| BraA07003380 | | -1.420141545 | 0.651730388 | -1.132853535 | -1.54973114 |
| BraA07003381 | | 0.75252361 | -0.442494549 | -0.225360882 | 0.191063557 |
| BraA07003382 | | 0.201122338 | 0.357612919 | -0.470389489 | 0.736347688 |
| BraA07003383 | | 0.293281522 | 1.457356046 | 0.893026998 | 1.156202085 |
| BraA07003384 | | 1.396321252 | 1.50418602 | 0.697415677 | 1.19019045 |
| BraA07003385 | | -1.548925943 | 0.03219445 | -0.229038138 | -0.96438502 |
| BraA07003386 | CRK2 | -1.546717648 | -2.225280011 | -2.738172422 | -2.887211662 |
| BraA07003387 | CRK3 | 0 | 0 | 0 | 0 |
| BraA07003388 | CRK3 | -0.793715559 | -0.784310255 | -1.593536027 | -2.365038286 |
| BraA07003389 | CRK3 | 1.826908473 | 2.426751153 | 2.209685867 | 1.217640824 |
| BraA07003390 | | 0.026281736 | -0.33655992 | -0.159299051 | -0.298703579 |
| BraA07003391 | SAV3,TAA1,WEI8 | -1.597270787 | -1.416715253 | 0.660911301 | -0.689654614 |
| BraA07003392 | AOAT2,GGT2 | -0.608424482 | -0.205140054 | -0.873433048 | -1.233039525 |
| BraA07003393 | | -5.271366891 | -4.337336223 | -3.1106792 | -3.36904766 |
| BraA07003394 | | -0.973678842 | -1.19934238 | -1.131341054 | -1.051711403 |
| BraA07003395 | | 0.051067891 | -0.739553972 | -0.332714828 | 0.140960235 |
| BraA07003396 | | 0.391096987 | -0.073165208 | 0.1532493 | 0.368824436 |
| BraA07003397 | | -0.776418693 | -0.372059689 | -0.321811553 | -0.547558585 |
| BraA07003398 | | -4.618084289 | 0.125319665 | -0.074484416 | 0.418528603 |
| BraA07003399 | | 0.522043117 | 0.866496802 | -0.652327805 | 0.111761626 |
| BraA07003400 | | -0.589848461 | -0.444621445 | -0.394446744 | -0.250784318 |
| BraA07003401 | | -0.399392448 | -0.295191876 | -0.304520226 | -0.450330743 |
| BraA07003402 | HWI1,PDLP5 | -3.047486063 | -2.1512071 | -1.095172912 | -0.707645635 |
| BraA07003403 | JAZ9,TIFY7 | 0.842342679 | -1.155301133 | -0.099256854 | -0.577081025 |
| BraA07003404 | ATGH9B1,CEL1,GH9B1 | 0.165385744 | -0.299628545 | 0.189041033 | 0.650050353 |
| BraA07003405 | | -0.939767618 | -1.139304732 | -1.928287456 | 0 |
| BraA07003406 | | 1.627920983 | 0.672535412 | -0.176586623 | 1.499371608 |
| BraA07003407 | CRR23 | 2.274000863 | -1.608211737 | -0.987398674 | -0.661148983 |

| | | | | | |
|---|---|---|---|---|---|
| BraA07003408 | | 0 | 0 | 0 | 0 |
| BraA07003409 | | 0.464092724 | 0.180508294 | 0.103336276 | 0.064731451 |
| BraA07003410 | | 0.263586987 | -0.042451453 | 0.04472756 | 0.18250086 |
| BraA07003411 | | -0.211888415 | -0.155377124 | -0.554953867 | -0.237782958 |
| BraA07003412 | | -3.830032692 | -0.844504622 | -0.934760768 | -0.702861042 |
| BraA07003413 | | 1.448098118 | -1.219240075 | -0.824896473 | -0.54553055 |
| BraA07003414 | | -1.718229051 | -1.402596142 | -1.213987814 | -1.861461537 |
| BraA07003415 | MLP28 | 0.198723481 | 0.230024079 | 0.748473333 | 1.229531326 |
| BraA07003416 | MLP28 | 0.295158933 | 0.514816504 | 1.156727139 | 1.11385311 |
| BraA07003417 | | -0.57522424 | 0 | 0 | 0.106781039 |
| BraA07003418 | | 0 | 0 | 0 | 0 |
| BraA07003419 | | 0 | 0 | 0 | 0 |
| BraA07003420 | MLP28 | -1.538281672 | 0.431128634 | 2.074169072 | 0.902004556 |
| BraA07003421 | MLP31 | 1.159196119 | 1.617444455 | 1.03859365 | 0.730192081 |
| BraA07003422 | CLE17 | 0.139344438 | 0.152961776 | 0.178020676 | 0.249408394 |
| BraA07003423 | | 0.206058316 | 0.096522441 | -0.090560527 | -0.127577439 |
| BraA07003424 | ATHB18,HB18 | -2.797301881 | 0.366202321 | -0.155464275 | -0.284784823 |
| BraA07003425 | ATPIN3,PIN3 | 0.013601076 | 0.009764622 | -0.33025515 | -0.455370732 |
| BraA07003426 | ATPIN3,PIN3 | 0 | 0 | 0 | 0 |
| BraA07003427 | | -0.22326579 | 0.093740502 | -0.621586734 | -0.279867605 |
| BraA07003428 | | -0.676925552 | 0.783655668 | 0.487191208 | -0.364203088 |
| BraA07003429 | | 0.249937766 | -0.364745086 | -0.502574346 | -0.446594792 |
| BraA07003430 | | -1.528579802 | -1.721196796 | -0.933278608 | -0.874715337 |
| BraA07003431 | | 0 | 0 | 0 | 0 |
| BraA07003432 | FAB1C | -0.479667378 | 0.312049556 | -0.196614684 | -0.016732541 |
| BraA07003433 | | -3.796921052 | 0 | 0 | 0 |
| BraA07003434 | | -3.098020169 | -0.980926322 | -1.198844441 | -0.936428889 |
| BraA07003435 | | -2.920049245 | -0.636615394 | -1.232316523 | -1.541379812 |
| BraA07003436 | ATMYBL2,MYBL2 | -0.251067533 | -2.006065662 | -2.850943807 | -2.55106084 |
| BraA07003437 | LPR2 | -1.217554056 | -0.716662705 | -0.796255657 | -0.66404822 |
| BraA07003438 | | 0 | -0.271623937 | -1.904938959 | 0 |
| BraA07003439 | | 2.112779537 | -0.914264936 | -0.517639564 | 0.122572606 |
| BraA07003440 | | -0.427128358 | -0.158176985 | -0.184139828 | -0.112291655 |
| BraA07003441 | PIN-LIKE2 | -0.358872136 | -0.359029898 | -0.566021872 | -0.676487148 |
| BraA07003442 | | -1.557831024 | -0.882021942 | -1.494958513 | -0.937078544 |
| BraA07003443 | | -1.696272408 | 0.786702117 | 0.041657339 | -1.14796511 |
| BraA07003444 | | 0 | 0 | 0 | 0 |
| BraA07003445 | | 0 | 0 | 0 | 0 |
| BraA07003446 | | 0 | -3.183334915 | -3.096441517 | -1.684969152 |
| BraA07003447 | | 0 | 0 | -8.161651192 | 0 |
| BraA07003448 | | 0 | -2.008732468 | 0 | 0 |
| BraA07003449 | | 0.16760469 | 0.010247568 | -0.04856831 | 0.00904417 |
| BraA07003450 | EBS1,PSL2,UGGT | 0.347834832 | 0.599589207 | 0.40880895 | 0.099073333 |
| BraA07003451 | | 0.923491845 | 0.065118144 | 0.628775269 | 0.574098517 |
| BraA07003452 | | -0.205882345 | 0.001529856 | 0.182996171 | 0.150577159 |
| BraA07003453 | | -1.089444667 | -1.155155471 | -1.339412315 | -1.1636263 |
| BraA07003454 | | 0 | 0 | 0 | 0 |
| BraA07003455 | ATWHY2,WHY2 | -0.546517102 | -0.399606547 | -0.1203403 | 0.069072615 |
| BraA07003456 | ATVPS52,POK,TTD8 | 0.430679838 | 0.32128757 | 0.378300724 | 0.159244023 |
